# Tissue-specific DNA methylation is conserved across human, mouse, and rat, and driven by primary sequence conservation

**DOI:** 10.1186/s12864-017-4115-6

**Published:** 2017-09-12

**Authors:** Jia Zhou, Renee L. Sears, Xiaoyun Xing, Bo Zhang, Daofeng Li, Nicole B. Rockweiler, Hyo Sik Jang, Mayank N.K. Choudhary, Hyung Joo Lee, Rebecca F. Lowdon, Jason Arand, Brianne Tabers, C. Charles Gu, Theodore J. Cicero, Ting Wang

**Affiliations:** 1 Department of Genetics, Center for Genome Sciences and Systems Biology, Washington University School of Medicine, St. Louis, MO USA; 2 Department of Psychiatry, Washington University School of Medicine, St. Louis, MO USA; 3 Division of Biostatistics, Washington University School of Medicine, St. Louis, MO USA

**Keywords:** DNA methylation, Epigenetic conservation, Tissue-specific, Comparative genomics, Comparative epigenomics

## Abstract

**Background:**

Uncovering mechanisms of epigenome evolution is an essential step towards understanding the evolution of different cellular phenotypes. While studies have confirmed DNA methylation as a conserved epigenetic mechanism in mammalian development, little is known about the conservation of tissue-specific genome-wide DNA methylation patterns.

**Results:**

Using a comparative epigenomics approach, we identified and compared the tissue-specific DNA methylation patterns of rat against those of mouse and human across three shared tissue types. We confirmed that tissue-specific differentially methylated regions are strongly associated with tissue-specific regulatory elements. Comparisons between species revealed that at a minimum 11-37% of tissue-specific DNA methylation patterns are conserved, a phenomenon that we define as epigenetic conservation. Conserved DNA methylation is accompanied by conservation of other epigenetic marks including histone modifications. Although a significant amount of locus-specific methylation is epigenetically conserved, the majority of tissue-specific DNA methylation is not conserved across the species and tissue types that we investigated. Examination of the genetic underpinning of epigenetic conservation suggests that primary sequence conservation is a driving force behind epigenetic conservation. In contrast, evolutionary dynamics of tissue-specific DNA methylation are best explained by the maintenance or turnover of binding sites for important transcription factors.

**Conclusions:**

Our study extends the limited literature of comparative epigenomics and suggests a new paradigm for epigenetic conservation without genetic conservation through analysis of transcription factor binding sites.

**Electronic supplementary material:**

The online version of this article (10.1186/s12864-017-4115-6) contains supplementary material, which is available to authorized users.

## Background

A fundamental yet unanswered question in biology is how epigenomes evolve. Comprised of an assortment of chemical modifications (including DNA methylation and histone modifications), the epigenome describes the genome-wide epigenetic landscape of a cell [1]. Within the context of cellular state, identical DNA sequences can diverge in their epigenetic patterning leading to differential gene expression, which is fundamental for the development of multicellular organisms [2]. Thus, a single genome shared among all cells has the potential to give rise to many epigenomes [3]. The information contained within a single genome must direct the creation of multiple epigenomes, but how the generation of these epigenomes is regulated and how epigenomes among different species relate to each other, remains largely undefined.

Comparative genomics studies genome evolution through the analysis of primary sequence divergence across species over time. Using this powerful method, many principles of genome evolution, adaptation, and function have been discovered [4], and functional regions of genomes identified [5, 6]. Thus, we hypothesize that by comparing epigenomes of multiple species in the context of their genomic sequences, one might deduce rules connecting genome evolution with epigenome evolution.

Pioneer studies in comparative epigenomics have begun to unveil the fundamental principles of epigenome evolution. For example, the genome-wide pattern of DNA methylation for certain genomic elements is conserved in vertebrates as well as plants [7, 8] suggesting that the regulatory roles of DNA methylation are conserved [9]. Using pluripotent stem cells of humans, mice, and pigs, Xiao et al. discovered strong epigenomic conservation in both rapidly evolving and slowly evolving DNA sequences, but not in neutrally evolving DNA sequences [10]. These conserved epigenomic modifications mark regulatory DNA [10, 11]. Using the Illumina HumanMethylation27 BeadChip microarray for human and chimpanzee liver, heart, and kidney samples, Pai et al. found that methylation variations were greater between tissues in the same species than between species for the same tissue [12]. Hernando-Herraez et al. compared DNA methylation patterns between humans and great apes using the Illumina HumanMethylation450 platform and found many genes with significantly altered methylation patterns. They discovered a positive relationship between the rate of coding variation and alterations of methylation at the promoter level [13]. Long et al. compared the location of non-methylated CpG islands (CGIs) in seven vertebrates and suggested that non-methylated regions are a conserved feature of vertebrate gene promoters [14]. In addition to DNA methylation, studies have associated the changes in other epigenetic marks between species with inter-species differential gene expression. One study examined the histone modification H3K4me3 in prefrontal cortex of human, chimpanzee, and macaque, and identified many sequences with human-specific enrichment or depletion [15]. Cain et al. investigated the contribution of H3K4me3 to regulatory differences between species and found strong evidence for conservation of H3K4me3 localization in the species examined. They estimated as much as 7% of inter-species gene expression differences could be explained by changes in H3K4me3 [16]. By comparing H3K27ac and H3K4me1 in livers across 20 mammalian species, Villar et al. demonstrated enhancer and slow promoter evolution [17]. Similarly, other assays that investigate differential chromatin states including Deoxyribonuclease I (DNase I) hypersensitive sites (DHSs), RNA polymerase II, and H3K4me1 have been found to be associated with gene expression among species [18, 19]. Recently, Prescott et al. compared epigenomic profiles of human and chimp induced pluripotent cell-derived cranial neural crest cells and revealed links between cis-regulatory divergence and quantitative expression differences of crucial neural crest regulators [20]. Together, these studies established the importance of epigenome conservation, and revealed that the relationship between genome conservation and epigenome conservation is not linear.

In this study, we address outstanding questions in comparative epigenomics: to what degree are tissue-specific epigenetic patterns conserved, and to what degree does an underlying genomic sequence account for a conserved epigenomic pattern? We focused our study on genome-wide DNA methylation. DNA methylation is a key epigenetic mechanism and plays critical roles in diverse biological processes such as X chromosome inactivation, repression of transposable elements (TEs), genomic imprinting, and tissue-specific gene expression [21, 22]. Disruption of normal DNA methylation is implicated in many diseases including cancer [23]. More recently, several studies have revealed that DNA methylation not only regulates promoters and CGIs, but plays a much larger role in regulation of tissue-specific expression [24–26]. However, the conservation of tissue and cell type-specific DNA methylation patterns across species has not been thoroughly assessed. This leaves a significant gap between our knowledge of genome evolution and epigenome evolution.

In our study, we compared DNA methylomes of multiple tissues (blood, brain, and sperm) from multiple species (human, mouse, and rat). We identified tissue-specific differentially methylated regions (tsDMRs) and compared their DNA methylation status as well as sequence conservation across species. We found that a significant proportion of tissue-specific DNA methylation is conserved. Conserved DNA methylation is associated with conservation of other epigenetic marks including histone modifications and conservation of primary genomic sequences. We found that the evolutionary dynamics of tissue-specific DNA methylation are best explained by maintenance or turnover of binding sites for important transcription factors (TFs). Our study extends the limited literature of Comparative Epigenomics and suggests a new paradigm for epigenetic conservation without genetic conservation through analysis of transcription factor binding sites (TFBSs).

## Results

### Up to 37% of rat tissue-specific differentially methylated regions are epigenetically conserved in mouse and human

**Fig. 1:**
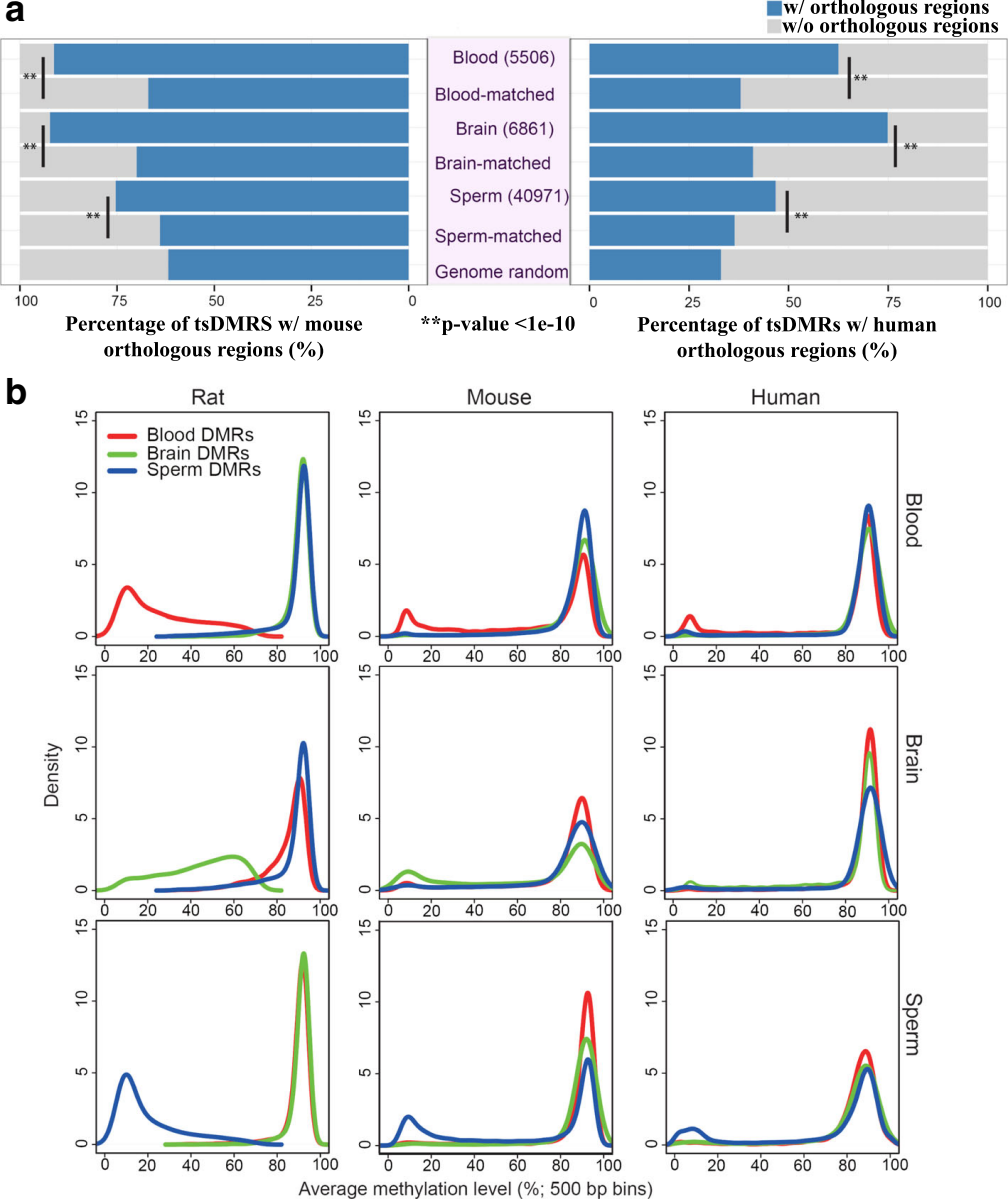
Rat tissue-specific DMRs (tsDMRs) and their orthologous regions in mouse and human. **a** Numbers of rat tissue-specific hypomethylated tsDMRs (*Methods*) (*middle* purple panel), and percentage of rat tsDMRs with orthologous regions in mouse (*left* panel) and human (*right* panel) genomes, respectively. A chi-square test was performed to obtain *p*-values by comparing tsDMRs with genomic annotation matched random control regions in each respective tissue type. *P*-values were corrected for multiple testing using the Benjamini–Hochberg FDR method. **b** Genome-wide methylation profile of rat tsDMRs and orthologous regions in mouse and human. The *left* column shows the methylation profiles of rat tsDMRs in each of the three rat tissue types; the *middle* column shows the methylation profiles of mouse orthologous regions of rat tsDMRs in each of the mouse tissue types; and the *right* column shows the methylation profile of human orthologous regions of rat tsDMRs in each of the human tissue types

We first produced DNA methylomes from three rat tissues (whole blood, whole brain, and sperm) using two complementary, sequencing-based technologies (Methylated DNA immunoprecipitation followed by sequencing (MeDIP-seq)) and methyl-sensitive restriction enzyme digestion followed by sequencing (MRE-seq)) [9, 27]. MRE signal is indicative of CpGs that are not methylated while MeDIP-seq indicates a methylated state for CpGs contained within the reads. Using computational tools these two data types can be integrated to call DMRs [26] and read out a methylation state for each CpG genome-wide [28]. For a complete description of data, please refer to Additional file 1: Table S1. As expected, the global distributions of CpG methylation across these three rat tissues overlap each other and reproduce the bimodal distribution seen in all vertebrates to date (Additional file 2: Figure S1). Previously published work supports global hypomethylation of sperm within the primate lineage [29]. However, our rat sperm methylation dataset better recapitulates global CpG DNA methylation levels seen in mouse sperm samples [30, 31]. Rat and mouse brain global CpG DNA methylation averages are similar to those previously published for human and mouse [32, 33]. Lastly, previous work has assayed many cells in the blood lineage in both human [33] and mouse [34] and we find the global average CpG DNA methylation levels of our mouse and rat blood samples to be in line with these averages. Using these data and recently developed computational algorithms [26, 28] (Methods), we defined tsDMRs for the three rat tissues. Previous research has indicated that the majority of tsDMRs are hypomethylated rather than hypermethylated in their respective tissues [26], so we focused our analysis on hypomethylated tsDMRs. In brief, tsDMRs were defined as 500 bp-sized genomic regions hypomethylated in one tissue, but hypermethylated in the other two tissues. In total we identified 5506 rat tsDMRs for blood, 6861 for brain, and 40,971 for sperm (Fig. 1a). Consistent with previous genome-wide DNA methylation profiling of other species [26], 91%-94% of tsDMRs were located in introns or intergenic regions (Additional file 2: Figure S2). Interestingly, even though global CpG methylation levels were comparable across the three rat tissues (Additional file 2: Figure S1A), there were considerably more tsDMRs in sperm, suggesting widespread local hypomethylation of the sperm methylome as compared to blood and brain methylomes. A higher proportion of sperm rat tsDMRs were in TEs when compared to blood and brain tsDMRs: 20% of sperm tsDMRs overlapped with TEs compared to 10% in blood and 5% in brain (Additional file 1: Table S2), including several TE subfamilies that were significantly enriched for hypomethylated DNA (Additional file 1: Table S3; Additional file 2: Figure S3, and Additional file 3). This result is consistent with a previous study showing repetitive elements in human sperm are frequently hypomethylated [29].

We next identified orthologous regions of rat tsDMRs in the genomes of mouse and human based on the pairwise chain files provided by the UCSC Genome Browser [35, 36] (Methods). For all subsequent analysis, only tsDMRs with orthologous regions are considered. Interestingly, rat tsDMRs were more likely to have orthologous counterparts in the mouse and human genomes than expected by chance (Fig. 1a). Note that we included both a genome-wide control and a control that matches the genomic distribution of tsDMRs within each tissue (Methods)(Fig. 1a and Additional file 2: Figure S3). Overall, 88% of rat tsDMRs were mapped to orthologous sequences in the mouse genome and 57% were mapped to orthologous sequences in the human genome, whereas the random expectations were 64% and 34% for mouse and human, respectively. The difference was statistically significant (Fig. 1a). Our strategy also allowed us to define regions that were orthologous in rat, mouse, and human (three-way orthologous regions) (Methods). We found 24,592 (46%) rat tsDMRs that were in three-way orthologous regions when 25% were expected by chance (and Additional file 1: Table S4). To avoid potential bias from DMRs within genic regions (exons and introns), we repeated this analysis using only tsDMRs within intergenic regions, and the results were similar (Additional file 1: Table S4). These data suggest that the epigenetic differences between tissues are more likely to be encoded by genomic sequences retained over evolutionary time than in species-specific sequences.

Having identified orthologous regions of rat tsDMRs in the genomes of mouse and human, we wanted to examine DNA methylation patterns of these regions in each respective species and in their matching tissue types. Thus, we generated and collected published DNA methylomes of samples with matching tissue types for mouse and human (Additional file 1: Table S1). As expected, rat tsDMRs exhibited strong hypomethylation in their respective tissue types (Fig. 1b, *left* column). Their orthologous regions in mouse and human also exhibited a tissue-specific pattern—they enriched for hypomethylation in tissues in which their rat counterparts were hypomethylated, but not in tissues in which their rat counterparts were hypermethylated (Fig. 1b, *middle and right* columns). However, the majority of the orthologous regions of rat tsDMRs in the other two species remain hypermethylated (Fig. 1b). Consequently, this data allowed us to estimate how often a rat tsDMR was epigenetically conserved (EC) in mouse, human, or both (Methods). We found that at least 27% (blood), 37% (brain), and 27% (sperm) of rat tsDMRs were EC in mouse, and at least 11% (blood), 13% (brain), and 11% (sperm) of rat tsDMRs were EC in human (Table 1). Among these, at least 6% (blood), 6% (brain), and 5% (sperm) of rat tsDMRs were EC across all three species. These results were all statistically significant (Table 1).

**Table 1:** tsDMRs by epigenetic conservation status (2-way analysis)

Rat-Mouse			
Tissue	EC	ENC	p-value
Blood	1477 (27%)	3583	<4.94e-324
Brain	2508 (37%)	4037	<4.94e-324
Sperm	11,149 (27%)	22,040	<4.94e-324
Rat-Human			
Blood	629 (11%)	2786	<4.94e-324
Brain	872 (13%)	4424	<4.94e-324
Sperm	4345 (11%)	15,263	2.52e-266

The percentage in parenthesis is calculated as the number of epigenetically conserved tsDMRs divided by the number of tsDMRs for each tissue type. A hypergeometric test was performed to determine if the epigenetic conservation was significant. The number of ‘observed’ epigenetically conserved tsDMRs is indicated in the ‘EC’ column. The number of ‘expected’ epigenetically conserved tsDMRs was determined by randomly selecting 40,000 rat regions and examining the number of these randomly picked regions that were epigenetically conserved between rat and mouse/human

A hypergeometric test was performed to obtain p-values. P-values were corrected for multiple testing using the Benjamini–Hochberg FDR method

*EC* Epigenetically conserved

*ENC* Epigenetically non-conserved

### Epigenetically conserved tsDMRs exhibit distinct genomic and epigenomic features as compared to epigenetically non-conserved tsDMRs

**Fig. 2:**
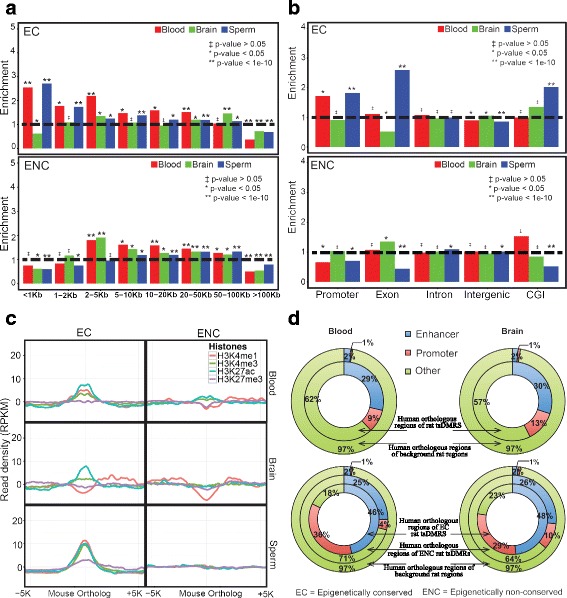
Epigenetically conserved rat tsDMRs and epigenetically non-conserved rat tsDMRs show distinct patterns. **a** Distribution of the distance between rat tsDMRs that are Epigenetically Conserved (EC) in mouse (*top* panel) and Epigenetically non-conserved (ENC) in mouse *(bottom* panel) to the nearest TSS. The horizontal dashed black line denotes no enrichment over the background. The background was a set of genomic distribution-matched rat regions. The y-axis represents the fold enrichment of orthologous rat tsDMRs over the background. A chi-square test was performed to generate the *p*-values. *P*-values were corrected for multiple testing using Benjamini–Hochberg FDR method. **b** Genomic distribution of rat tsDMRs that are EC in mouse (*top* panel) and ENC in mouse (*bottom* panel). The background regions were chosen as described in (**a**). A chi-square test was performed to generate the *p*-values. P-values were corrected for multiple testing using the Benjamini–Hochberg FDR method. **c** Average histone modification signal density at 50 bp resolution over a 10-kb window centered on mouse orthologous regions of rat tsDMRs that are EC (*left* column) and ENC (*right* column) in mouse blood (*top* row), mouse brain (*middle* row), and mouse sperm (*bottom* row). **d** ChromHMM regulatory function annotation of human orthologous regions of rat blood tsDMRs (*top left* panel), rat brain tsDMRs (*top right* panel), EC and non-conserved rat blood tsDMRs (*bottom left* panel), and EC and non-conserved rat brain tsDMRs (*bottom right* panel). In all panels, the annotation of the human orthologous regions for randomly chosen rat regions was included. The background regions were chosen the same way as described in (**a**) except that human instead of mouse orthologous regions were used

Having categorized rat tsDMRs with orthologous regions based on their conserved epigenetic pattern in mouse and human (EC vs. epigenetically non-conserved (ENC)), we investigated genomic and epigenomic features of each tsDMR category. First, we calculated the distance between each rat tsDMR and the nearest gene transcription start site (TSS) to create a distribution for each tissue and species. When we compared rat and mouse, we found that EC tsDMRs of blood and sperm, but not those of brain, were enriched in regions near TSSs over the randomly selected genomic feature-matched background (Fig. 2a). In contrast, we found a depletion of ENC tsDMRs near the TSS compared to the genome feature-matched background. Instead, ENC tsDMRs showed enrichment 2-5Kb from the TSS for blood and brain and at all other distances further from the TSS (other than >100 Kb) for all three tissues (Fig. 2a). A similar pattern was observed for the rat and human comparison (Additional file 2: Figure S5A). Direct comparison of distance from the TSS to either EC or ENC tsDMRs was performed and in all, except the rat-mouse EC to ENC comparison, rat regions that were EC were closer to TSSs than their ENC counterparts (Additional file 1: Table S5). These results were further confirmed by an analysis of tsDMRs’ association with different genomic features (including promoters, exons, introns, intergenic regions, and CGIs) (Fig. 2B and Additional file 2: Figure S5B). The EC mouse and human orthologous regions also showed increased proximity to known TSSs. No such enrichment was found in the ENC group (Additional file 2: Figure S6). Both, EC and ENC tsDMRs were more enriched for non-CGI promoters than CGI containing promoters, but the fold enrichment of EC tsDMRs over the background for non-CGI promoters was significantly higher than that of ENC tsDMRs (Additional file 2: Figure S7). Since genes associated with non-CGI promoters tend to have more tissue- or developmental stage-restricted expression patterns [37], our result is consistent with the expectation that the epigenetic patterning in these genes would be shared in a tissue-specific manner across evolutionary time.

We next asked if EC tsDMRs and ENC tsDMRs have different chromatin signatures. To this end, we collected published histone modification profiles (H3K4me1, H3K4me3, H3K27ac, and H3K27me3) for comparable mouse and human tissues (Additional file 1: Table S1) and computed the fractions of tsDMRs that overlap with each histone modification mark (Table 2). We found a striking difference between EC and non-conserved tsDMRs when analyzed in the context of histone marks that indicate transcriptional activity (Table 2).

**Table 2:** Summary of overlap between histone mark peaks and mouse and human orthologous regions of rat tsDMRs

Mouse					
Blood					
	# tsDMRs	H3K4me1	H3K4me3	H3K27ac	H3K27me3
EC	1477	181	146	193	5
ENC	3583	282	86	145	16
p-value		8.77E-07	5.67E-31	1.54E-31	0.587
Brain					
EC	2508	826	70	551	18
ENC	4037	801	44	269	19
p-value		9.85E-33	3.15E-07	6.73E-74	0.195
Sperm					
EC	11,149	3690	1889	559	334
ENC	22,040	1941	555	314	155
p-value		0	0	5.62E-83	3.02E-60
Human					
Blood					
EC	629	311	138	221	24
ENC	2786	346	77	197	117
p-value		1.84E-100	1.57E-71	8.21E-84	0.662
Brain					
EC	872	272	207	250	33
ENC	4424	992	429	710	180
p-value		2.82E-08	2.09E-31	9.40E-19	0.696

Overlap between a histone mark peak and a mouse/human orthologous region was defined if the orthologous region contained the summit of the histone mark peak

A chi-square test was performed to obtain p-values. P-values were corrected for multiple testing using Benjamini–Hochberg FDR method

The EC tsDMRs exhibited much higher enrichment of transcriptionally active histone marks (H3K4me1, H3K4me3, and H3K27ac) in their respective tissue as compared to the ENC tsDMRs. To illustrate this, we plotted the average histone modification signals over rat tsDMRs in orthologous regions in the mouse genome (Fig. 2c) and in the human genome (Additional file 2: Figure S8). For example, in mouse blood, EC blood tsDMRs showed enrichment, for both enhancer (H3K4me1 and H3K27ac) and promoter (H3K4me3) marks, with stronger enrichment for enhancers. In contrast, in mouse brain, EC brain tsDMRs were enriched mainly for active enhancers (i.e. H3K27ac). EC sperm tsDMRs also showed relative enrichment for both enhancer and promoter histone marks in mouse sperm.

The ENC tsDMRs did not enrich for any of the active histone marks. In addition, no enrichment was observed for the repressive mark H3K27me3 in either EC tsDMRs or ENC tsDMRs (Fig. 2c). Examination of human histone modification data for the blood and brain revealed a somewhat similar pattern (Additional file 2: Figure S8). This pattern was further confirmed by examining the chromatin state annotation of the human orthologous regions of the rat tsDMRs, using chromHMM [38]. Using chromatin states defined by the nine ENCODE cell lines [39], we found that human orthologous regions of rat tsDMRs (HO-tsDMRs) were enriched for regulatory elements including enhancers and promoters. The enrichment was much more dramatic in EC regions than ENC regions (Fig. 2d). Taken together, this data underscores the functional potential of epigenetic conservation.

### Epigenetic conservation is associated with genetic conservation

**Fig. 3:**
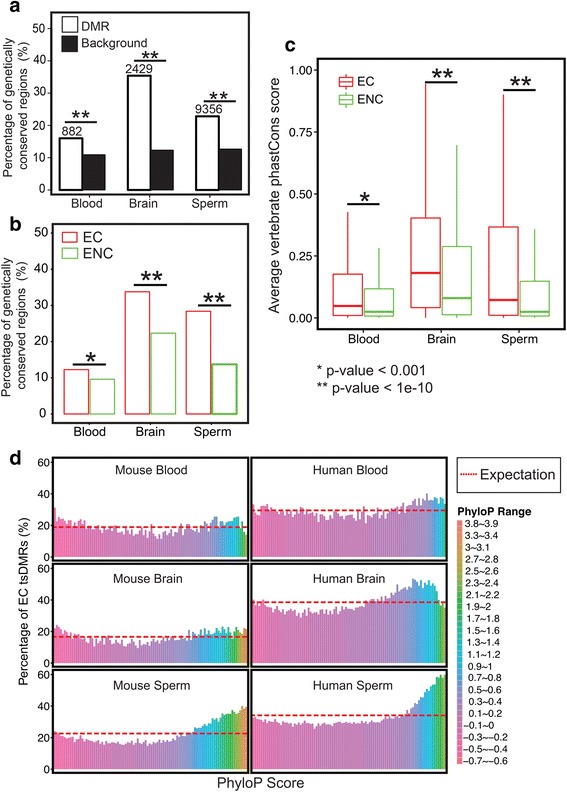
Epigenetically conserved tsDMRs and epigenetically non-conserved tsDMRs show distinct genetic conservation. **a** Percentage of rat tsDMRs that are genetically conserved. A pre-defined list of rat-conserved elements was determined using the Hidden Markov model from the UCSC Genome Browser. A rat tsDMR was defined as “genetically conserved” if at least 20% of the rat tsDMR region overlapped with genetically conserved elements (Methods). The number of genetically conserved tsDMRs is indicated above the bars for each tissue type. For each tissue type, a genomic annotation matched random control set was chosen. A chi-square test was performed to obtain *p*-values. *P*-values were corrected for multiple testing using the Benjamini–Hochberg FDR method. **b** Percentage of EC rat tsDMRs mapped in mouse that overlap with genetically conserved rat elements (in red) and the percentage of ENC rat tsDMRs mapped in mouse that overlap with genetically conserved rat elements (in green). A chi-square test was performed to obtain the p-values. P-values were corrected for multiple testing using the Benjamini–Hochberg FDR method. **c** Distribution of phastCons scores of EC rat tsDMRs and ENC rat tsDMRs in mouse. A Wilcoxon test was performed to obtain p-values. P-values were corrected for multiple testing using the Benjamini–Hochberg FDR method. **d** Epigenetic conservation (Y-axis) and genetic conservation as defined by PhyloP scores (X-axis) (Methods)

To determine the driving forces that maintain epigenetic conservation, we examined the overall sequence conservation of rat tsDMRs. First, we asked how often tsDMRs overlap with genetically conserved elements defined by the UCSC phastCons nine-way vertebrate elements track [40]. Compared to feature-matched expectation, all categories of tsDMRs were highly enriched for conserved elements (Fig. 3a). Strikingly, EC tsDMRs contained statistically more genetically conserved elements than ENC tsDMRs did (Fig. 3b). This pattern was substantiated by directly comparing the rat phastCons scores of EC tsDMRs and ENC tsDMRs (Fig. 3c) [40]: EC tsDMRs exhibited higher sequence conservation than ENC tsDMRs (Fig. 3c). Concerned that genomic sequences associated with genes might be conserved for other reasons (i.e. coding potential), we repeated this analysis using only tsDMRs within intergenic regions and the results were similar (Additional file 2: Figure S9**)**. Thus far, our analysis revealed that epigenetic conservation is strongly associated with genetic conservation, but that genetic conservation does not account for all epigenetic conservation.

To further elucidate the relationship between genetic conservation and epigenetic conservation, we ranked tsDMRs based on their average phyloP scores [41] and partitioned them into groups of 100 (Methods). phyloP scores can be interpreted as probability of selection and conservation, where positive values represent conservation and negative values mean fast-evolving, thus allowing for detection of sites under negative or positive selection. For any given phyloP score range, we asked if tsDMRs were more or less likely to be EC (Fig. 3d). This analysis confirmed that genetically conserved tsDMRs, i.e., those whose DNA sequences were under negative selection, were more likely to be EC. Interestingly, tsDMRs under positive selection had a slightly increased likelihood of being EC when compared to those with neutrally evolving sequences.

### Epigenetic conservation can be explained by conservation of TF binding sites

Our analyses thus far have suggested that primary sequence conservation is strongly associated with epigenetic conservation. However, the association is far from linear as there are many regions with discordant genetic and epigenetic conservation, i.e., genetically conserved but ENC tsDMRs, or genetically non-conserved but EC tsDMRs. Previous studies suggested that tissue-specific DMRs are regulatory elements that are enriched for TF binding motifs [42, 43]. Thus, tissue-specific DNA binding factors could be another force that drives epigenetic conservation. To understand how the evolution of TFBSs might have regulated tissue specification via regulating DNA methylation, we investigated the association between tissue-specific TFBSs and epigenetic conservation of tsDMRs.

**Fig. 4:**
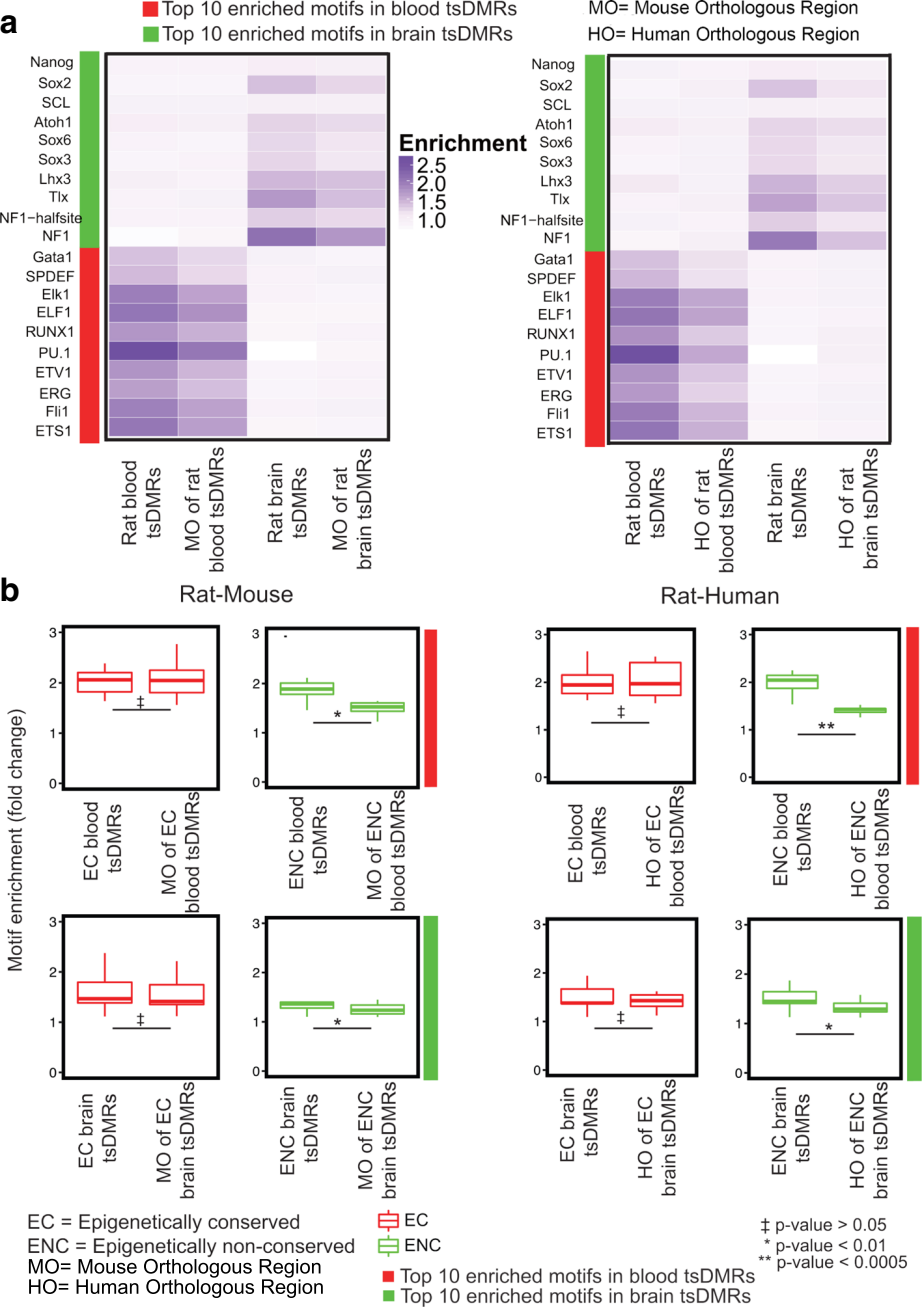
TF motif analysis of rat tsDMRs and their orthologous regions in mouse and human. **a** Heatmaps representing the enrichment of transcription factor binding motifs for brain and blood tsDMRs the orthologous regions of these tsDMRs. *Left* panel: motif enrichment for rat-mouse comparison; *right* panel: motif enrichment for rat-human comparison. **b** Motif enrichment (fold change) of the top 10 TFBSs in rat blood (*top* row) and rat brain (*bottom* row) (as defined by the HOMER enrichment), separated by their epigenetic conservation status (Methods). *First two* columns: rat-mouse comparison; *last two* columns: rat-human comparison. A t-test was performed to obtain p-values. P-values were corrected for multiple testing using the Benjamini–Hochberg FDR method

We first asked if tsDMRs were enriched for tissue-specific TF binding motifs over the GC% content match background sequences that were randomly selected by the motif discovery algorithm. Using HOMER [44], we identified the most significantly over-represented motifs within rat blood, brain, and sperm tsDMRs (Methods). Indeed, many of the identified motifs were associated with TFs relevant to the specific tissues (Fig. 4a). For example, the most enriched sequence motifs in blood tsDMRs were those of the ETS TF family, which are master regulators of hematopoiesis [45] and involved in the pathology of diseases of the blood [46]. Specific factors include PU.1, which activates gene expression during myeloid and B-lymphoid cell development [47, 48] and Erg, which is required for platelet adhesion and regulation of hematopoiesis [49]. Similarly, in brain tsDMRs, motifs of TFs important for neuronal functions were highly enriched. Among these, Lhx3 is required for pituitary development and motor neuron specification [50, 51] and NF1 is essential in specifying brain-specific gene expression [52]. The pattern in sperm was not as strong as those in blood and brain, presumably due to the relative lack of annotated sperm-specific TF binding motifs.

We next assessed if these TF motifs were also enriched in the orthologous sequences of tsDMRs in mouse and human. Overall, we observed that the same sequence motifs were enriched in the orthologous sequences of respective rat tsDMRs in both mouse and human, but to a lesser degree than in the rat tsDMRs (Fig. 4a). Interestingly, when we compared the enrichment of the same set of motifs over the Homer selected background in orthologous sequences that were EC versus those that were ENC, we found high enrichment levels in the EC orthologous sequences, but low enrichment levels in the ENC orthologous sequences (Fig. 4b). Taken together, these data suggest that TF binding motifs are strongly correlated with epigenetic conservation [53].

### Evolutionary dynamics of tsDMRs

Our epigenomic data spans three common tissue types across three mammalian species. This data not only allows us to identify EC tsDMRs, but also gives us an opportunity to investigate the evolutionary dynamics of some of these tsDMRs. We focused our analysis on rat tsDMRs for which we can identify three-way orthologous regions in mouse and human (Methods), and examined the epigenomic configuration of these regions in the context of the three species.

Overall, we identified 24,592 rat tsDMRs that shared three-way orthology with mouse and human. Of these, 2796 were EC across all three species (Group 1, blood: 309 out of 2859; brain: 402 out of 4746; sperm: 2085 out of 16,987). 6205 were EC between rat and mouse, but not human (Group 2, blood: 611; brain: 1551; sperm: 4043), and 2114 were EC between rat and human, but not mouse (Group 3, blood: 235; brain: 360; sperm: 1519). The remaining 11,972 tsDMRs were rat-specific and not EC in either mouse or human (Group 4) (Table 3).

**Table 3:** Rat tsDMRs with various epigenetic conservation status (3-way analysis)

Tissue	RMH EC	RM EC	RH EC	RMH ENC
Blood	309	611	235	1576
Brain	402	1551	360	2251
Sperm	2085	4043	1519	8145

*RMH EC* Epigenetically conserved rat tsDMRs across rat, mouse, and human;

*RM EC* Epigenetically conserved rat tsDMRs in rat and mouse, but not human;

*RH EC* Epigenetically conserved rat tsDMRs in rat and human, but not mouse;

*RMH ENC* Rat tsDMRs that are epigenetically not conserved in either mouse or human

We compared the genomic distribution, sequence conservation, histone modification, and TFBS enrichment across these four groups of tsDMRs (Additional file 2: Figures S10-S13). The results recapitulated the patterns we observed from the pairwise comparisons. In general, when compared to ENC tsDMRs, EC tsDMRs were closer to TSSs, were more enriched for active histone marks, contained more conserved sequences, and were more enriched for binding motifs of relevant TFs (Additional file 2: Figures S10-S13).

**Fig. 5:**
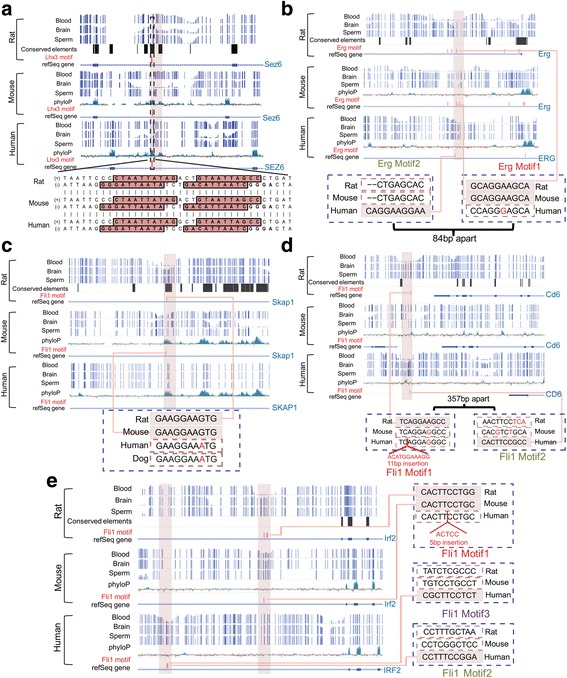
Evolutionary dynamics of tsDMRs and transcription factor binding sites. WashU EpiGenome Browser [80] views of tsDMRs in rat and the orthologous regions in mouse and human. The following tracks are displayed: *rat*: rat DNA methylation (methylCRF) for blood, brain, and sperm, phastCons conserved elements, TFBSs, and refSeq gene annotation; *mouse*: mouse DNA methylation (methylCRF/WGBS) for blood, brain, and sperm, 30-way vertebrate phyloP score, TFBSs, and refSeq gene annotation; and *human*: human DNA methylation (methylCRF/WGBS) for blood, brain, and sperm, 46-way vertebrate phyloP score, TFBSs, and refSeq gene annotation. Rat tsDMRs and their orthologous regions in mouse and human are highlighted in pink rectangles. The predicted TF motifs are represented by the vertical red bars. **a** Genome browser view of a rat brain DMR and its mouse and human orthologous regions located in the intronic region of the Sez6 gene. **b** Genome browser view of a rat blood DMR and its mouse and human orthologous regions located in an intronic region of the Erg gene. **c** Genome browser view of a rat blood DMR and its mouse and human orthologous regions located in an intronic region of the Skap1 gene. **d** Genome browser view of a rat blood DMR and its orthologous regions in mouse and human located in the downstream region of the Cd6 gene. **e** Genome browser view of a rat blood DMR and its mouse and human orthologous regions located in an intronic region of Irf2 gene

These comparisons allowed us to examine patterns of evolutionary change within tsDMRs (Fig. 5). Of the 2796 EC tsDMRs across the three species, 1661 were also genetically conserved as defined by PhastCons conserved elements (Methods). One such example was a brain specific hypomethylated DMR located in the fourth intron of Sez6 in the rat genome (chr10: 64,007,000-64,007,500). Sez6 is a brain-specific gene that encodes a protein related to seizures [54]. The orthologous regions of this tsDMR in mouse and human (Additional file 1: Table S6) were EC in each species, exhibiting hypomethylation in brain, and hypermethylation in blood and sperm. This brain tsDMR was located 35 kb downstream of the TSS in rat and mouse, and 38 kb in human, and contained four predicted Lhx3 motifs in all three species. Motif sequence alignment suggested that the core motif, CTAATTAATT, was indeed conserved across the species. Thus, this analysis put Lhx3, a TF essential in brain function [51], upstream of Sez6. Studies using mouse primary neurons associated Sez6 with pentylenetetrazol-induced bursting activity, an attribute of neurons undergoing epileptic discharges [54, 55] Sez6 mutation is also associated with febrile seizures in children [56]. These data are consistent with the hypothesis that brain tsDMRs that are epigenetically and genetically conserved should be functionally important in the tissue of interest (Fig. 5a **;** Additional file 1: Table S6).

There were 1135 tsDMRs that were EC, but genetically not conserved. Our genomic analysis suggested that conservation of TF binding could be a driving force of this phenomenon. Taking the Erg gene as an example, we identified an EC, blood-specific hypomethylated DMR 3 kb downstream of Erg gene’s TSS (Additional file 1: Table S6). Sequences of this tsDMR were not conserved across the three species, as indicated by the lack of high phyloP scores and lack of conserved elements (phastCons). Erg is an important regulator of differentiation for early hematopoietic cells [57]. Interestingly, we found that in all three species, there was an Erg motif within the EC tsDMR. However, the position of this motif shifted between rodents and human. A close examination of the primary sequence alignment revealed that rat and mouse shared a conserved Erg motif (consensus: ACAGGAAGTG), but in human, an A➔G mismatch within the orthologous region of the rat motif sequence destroyed the Erg binding motif. Surprisingly, the human tsDMR had an Erg motif 84 bp upstream from the rodent motif (Fig. 5b **;** Additional file 1: Table S6). This new binding site suggests an evolutionary event of TFBS turnover. Many studies have suggested that binding site turnover is a common, wide-spread phenomenon of gene regulatory network evolution [58–60]. We hypothesize that binding site turnover potentially helped maintain the conserved epigenetic pattern, despite the lack of primary sequence conservation.

Of the 6205 tsDMRs that were EC in rat and mouse, but not human, 2915 were genetically conserved. Similarly, of the 2114 tsDMRs that were EC in rat and human, but not mouse, 864 were genetically conserved. We sought to explain some of these phenomena.

We identified chr10:85,389,000-85,389,500 as a rat blood EC tsDMR in mouse. This region was in the intron of the Skap1 gene, which encodes a T-cell adaptor protein that regulates T-cell receptor signaling [61]. However, the human orthologous region was consistently highly methylated across the three tissues. The rodent tsDMR contained a conserved Fli1 motif, but the human orthologous region of the motif had a G➔A mismatch, which destroyed the motif. By including the orthologous dog sequence as an outgroup, we found that the Fli1 motif is possibly gained (by substitution A➔G) within the rodent branch, suggesting that this tsDMR represents a rodent-specific event likely driven by the Fli1 motif (Fig. 5c **;** Additional file 1: Table S6).

We also identified chr1:213,289,000-213,289,500 as a rat blood tsDMR. This region is 40 kb downstream of the TSS of the Cd6 gene [62]. The human orthologous region was also a blood tsDMR, but the mouse orthologous region was consistently hypermethylated in all three tissues. Congruent with this pattern, we found a Fli1 motif in both rat and human orthologous tsDMRs, but not in the mouse orthologous region. The mouse and human sequence elements that aligned to the rat Fli1 motif (TCAGGAAGCC) both had the same substitution that disrupted the motif. The most parsimonious explanation was that the motif was a rat-specific gain. 357 bp away from this site, but still within the human tsDMR, there was a Fli1 motif. Neither the sequences in the orthologous region in rat or mouse matched the Fli1 motif consensus sequence. Thus, the epigenetic dynamics of this tsDMR did not correlate with sequence conservation, but rather they were correlated with a species-specific TFBS (Fig. 5d **;** Additional file 1: Table S6).

Some of these ENC tsDMRs were associated with not only TFBS turnover, but also possibly tsDMR turnover. An interesting example was the blood tsDMR (rat, chr16:48,707,500-48,708,000) in the intron of the Irf2 gene. Irf2 encodes interferon regulatory factor 2, a member of the interferon regulatory TF (IRF) family. The IRF family plays an important role in the immune system [63]. The orthologous region in mouse was also identified as a blood DMR, but in human, the orthologous region was hypermethylated in all three tissues. Motif analysis revealed that the rat and mouse orthologs shared the Fli1 motif, but the human orthologous sequence contained a 5 bp insertion, disrupting the Fli1 motif. By examining the surrounding regions in the human genome, we found a human blood tsDMR approximately 6 kb upstream of the ENC human orthologous region of the rat tsDMR. This human tsDMR contained two predicted Fli1 motifs. Interestingly, the orthologous region of this human blood tsDMR in rat and mouse were not blood tsDMRs and did not have the Fli1 motif (Fig. 5e **;** Additional file 1: Table S6). It is possible that the human tsDMR and rodent tsDMR are functionally equivalent. This example is suggestive of a tsDMR turnover event that was correlated with a TFBS turnover event.

## Discussion

Dynamic changes of DNA methylation play a key role in development and differentiation by defining tissue and cell type-specific epigenomes [64]. Although the mechanism of DNA methylation mediated epigenetic regulation is conserved across vertebrates [8], the genome-wide conservation pattern of tissue-specific DNA methylation has not been thoroughly investigated. The genetic mechanism underlying epigenetic conservation is poorly understood.

Our study extends the limited literature of Comparative Epigenomics [14] and suggests a new paradigm for epigenetic conservation without genetic conservation through analysis of transcription factor binding sites. Previous comparative epigenomics studies investigated conserved epigenetic modifications of one tissue or cell type across species [10]. We expanded upon this framework and defined epigenetic conservation as conserved tissue-specific DNA methylation. Thus, our study is a cross-species comparison of the differences among epigenomes representing different tissue types.

By focusing on the dynamics of the DNA methylomes, we added strength to the notion that specific elements are important for specific tissue types. Consistent with recent discoveries [26, 43], these tissue-specific regulatory elements were often themselves tsDMRs. Compared to random genomic sequences, these tsDMRs were more likely to have orthologous counterparts in the three species we studied, indicating the importance of retaining these sequences. Analyses of the conservation pattern of these tsDMRs revealed several important principles.

First, tsDMRs were more likely to be EC than expected by chance. This was perhaps not too surprising, because tissue-specific gene expression is known to be conserved between species [65]. However, to our knowledge, this is the first study to define and quantify such epigenetic conservation. For example, we found that at a minimum between 11% and 37% of rat tsDMRs were EC in human or mouse depending on the tissue of interest. EC tsDMRs also exhibited conserved histone modifications, consistently supporting the regulatory roles of these tsDMRs. Perhaps most surprising was the result that the majority of tsDMRs were not EC. This is consistent with the idea that regulatory regions undergo rapid turnover, but could be confounded by differing cell type frequencies and developmental time points across the samples used in this analysis.

Compared to ENC tsDMRs, EC tsDMRs were more likely to also be genetically conserved. This result suggests that sequence conservation was likely a primary driver of epigenetic conservation. Fast evolving sequences were also more likely to be EC. Sequence analysis discovered that EC tsDMRs were strongly enriched for binding motifs of TFs relevant to that tissue type, more so than tsDMRs that were not EC. This result suggests that TFBS played crucial roles in determining epigenetic conservation, consistent with the discovery that sequence-specific DNA binding factors shape the landscape of DNA methylomes [42, 43] as well as DHSs [66].

Importantly, TFBS turnover seemed to be associated with evolutionary dynamics of epigenetic conservation. Some tsDMRs were EC, but not genetically conserved. These tsDMRs often contained a binding site of a relevant TF, although the binding sites themselves did not align at orthologous positions between species. However, the presence of the binding site was indeed a conserved genetic event. In contrast, some tsDMRs were genetically conserved, but were not EC. Close examination of the primary sequence alignments revealed interesting examples in which genetic changes, that did not interrupt the overall conservation level, destroyed a binding site for a relevant TF. This hypothesis is consistent with a recent study showing that turnover of TF recognition elements was associated with the repurposing of DHSs [66].

Our study has several limitations. Analysis of TFBS turnover is limited by two factors: first, all TFBSs are based on motif prediction; second, our knowledge on binding specificity of many TFs is quite incomplete. These inhibit us from addressing causality, i.e., whether epigenetic conservation is indeed ‘determined’ by TF motif conservation or if it is just incidental to these factors, in this study. We expect future experiments will eventually establish causality. Mapping of orthologous tsDMRs was performed with a liftover requirement of 50% non-reciprocal overlap, which could mask small regions of high conservation within larger DMR blocks. For this reason and others below, we believe all percentages presented throughout the paper represent an underestimation of the true epigenetic conservation of tsDMRs across the species studied. Additionally, a short highly conserved element may lie within a tsDMR and thus our annotation of genetically conserved tsDMRs may also be an underestimate. Although MRE-seq is insensitive to non-CG methylation, MeDIP-seq is sensitive to this type of methylation. Since up to 25% of neuronal methylation may be in a non-CG context [67], the proportion of non-CG methylation could contribute to the genomic distribution of our brain DMRs and can not be directly accounted for in this analysis. The tissue samples were heterogeneous in cell type composition and our brain samples are not exactly matched for developmental stage. Thus, our analysis could not distinguish differences at tissue level from differences due to different cell type compositions or developmental stage. We believe these limitations could deflate the number of EC tsDMRs observed and inflate the number of ENC tsDMRs. However, by including three tissue types we were able to characterize differences in epigenetic conservation between tissues, which have previously been shown to be greater than differences between cell-types comprising any tissue [26]. For example, the genomic distribution of brain tsDMRs is different from that of blood and sperm. While for both blood and sperm we observed that tsDMRs tend to enrich for being closer to TSS of known genes, for brain we did not observe this pattern. Our histone modification analysis suggested that blood and sperm tsDMRs are enriched for both active enhancers and promoters, while brain is more enriched for active enhancer marks. Furthermore, brain tsDMRs show more intermediate methylation in brain, while the tsDMRs in the other two tissues show low methylation in their respective tissue types. All of these data are in agreement with the hypothesis that the tissue specificity of the brain is mainly determined by enhancer activity. This is consistent with previous reports [68] that brain specific DMRs are enriched for enhancers and depleted for CGIs and CGI-promoters. Our conclusions regarding genome-wide brain specific DNA methylation should not be greatly influenced by including different cell types from the brain. However, in our future studies, we expect to assess epigenetic conservation of specific cell types across species. Lastly, our study defined tsDMRs as regions of low methylation, since hypomethylation has previously been associated with tissue-specific gene ontology functions and accessible chromatin [26, 42, 69]. However within this framework hypermethylated regions that could signify regulatory regions deactivated in a tissue-specific manner are not accounted for in this analysis. Additionally, it is well know that certain transcription factors interact with methylated CpGs [70] with more recent studies examining this interaction for factors without a methyl-CpG binding domain [71], thus hypermethylated regions could also signify regions with tissue-specific transcription factor interactions. Taken together, our study established that tissue-specific DNA methylation was conserved across species, and this epigenetic conservation was largely driven by genetic sequence conservation, including conserved TFBS. Our work provides a new paradigm for comparative epigenomics studies. We envision that future studies will include DNA methylomes from additional cell/tissue types and species, allowing us to better model epigenome evolution in the context of genome evolution. Such an understanding will contribute theories of how cellular differentiation evolved and how the epigenome contributes to cellular phenotype and identity.

## Conclusions

DNA methylation underlies cell type-specificity through its regulation of gene expression. Although it is known that the overall pattern of genome-wide DNA methylation is conserved across many species, little is known about the genetic underpinnings of this epigenetic conservation. Here we compare genome-wide DNA methylomes of three tissues across three species. We find that orthologous regions of genomic regions with tissue-specific hypomethylation are much more likely to share this pattern than expected by chance and term this “epigenetic conservation”. Regions with epigenetic conservation are strongly associated with gene regulatory elements and active chromatin modifications. While primary sequence conservation underlies epigenetic conservation, maintenance and turnover of transcription factor binding sites is likely the driving force behind tissue-specific DNA methylation.

## Methods

### Animals

All experiments were approved by the Institutional Animal Care and Use Committee of Washington University in St. Louis and conducted in accordance with the National Institutes of Health Guidelines for the Care and Use of Laboratory Animals. For each of the three tissues, pooled samples from fifteen adult rats (60 days old) were obtained.

### Processing MeDIP-seq and MRE-seq data

The reads were aligned to rn4, mm9, and hg19 for rat, mouse, and human, respectively with BWA [72],and were further processed by methylQA [73] (http://methylqa.sourceforge.net/). MRE reads were normalized to account for differences in enzyme efficiency. Scoring consisted of tabulating reads with a CpG at each fragment end [9].

### Processing histone modification data

H3K4me1, H3K4me3, H3K27ac and H3K27me3 ChIP-seq data for relevant tissue types were downloaded from the Gene Expression Omnibus (GEO) database [74]. Mapped read density was generated from aligned sequencing reads using in-house Perl scripts. Read density overlapping tsDMRs and the extensions to their upstream/downstream regions were extracted at 50-bp resolution as Reads Per Kilobase of transcript per Million mapped reads (RPKM) values.

### Processing whole-genome bisulfite sequencing data

Whole-genome bisulfite sequencing (WGBS) data for mouse and human sperm were downloaded from the GEO database (Mouse sperm accession number: GSE49623; Human sperm accession number: GSE30340). Downloaded human WGBS data was in hg18, thus we converted the aligned data to hg19 coordinates using the UCSC Liftover package [75]. If the new coordinates in the hg19 assembly overlapped with hg19 CpG sites, they were retained for downstream analysis.

### Defining tissue-specific hypomethylated DMRs

The M&M package [26] was used to identify DMRs between tissue types (see details in Additional file 3). All DMRs are based on a default genomic window size of 500 bp, which has been previously used in many studies [24–26]. The selection of DMRs for downstream analysis was based on the following criteria: 1) DMR q-values were <1e-5 and 2) for each tissue type, only hypomethylated DMRs (as defined below) in that tissue were retained.

MethylCRF [28] scores predicted by the methylCRF algorithm served as another layer of filtering criteria. Scores were first averaged over all CpGs within a M&M DMR and M&M DMRs that meet either the following criteria were retained for the downstream analysis: (1) the methylCRF score in the tissue type in which the M&M DMR was identified as hypomethylated should be <0.3, and the methylCRF score in the other two tissue types should be both ≥0.3; or (2) the methylCRF score in the tissue type in which the M&M DMR was identified as hypomethylated is ≥0.3 and ≤0.7 (intermediately methylated), and the methylCRF in the other two tissue types are both >0.7 (hypermethylated).

### Obtaining genomic distribution-matched control set

The rat (rn4) genome was divided into 500 bp non-overlapping windows, and for each window, genomic feature association was obtained. Similarly, the genomic feature associations of tsDMRs were obtained. For each set of tsDMRs associated with a certain genomic feature, the same number of rat 500 bp windows was selected from a random genomic feature matched set. Each rat tsDMR or 500 bp region is defined as being associated with a genomic feature (promoter, exon, 3’utr, 5’utr, CGI, etc.) only if more than 50% of the nucleotides in the tsDMR/500 bp region overlapped with that genomic feature.

### Mapping orthologous regions

The rat (rn4) genome was divided into approximately 5 million 500 bp non-overlapping regions, and rat tsDMRs were identified. The orthologous regions of rat tsDMRs in mouse (mm9) and human (hg19) genomes were identified using UCSC liftover chain files. We required at least 50% non-reciprocal overlap of the original sequence in rat to the species (mouse or human) of interest with an upper bound of 1000 bp for the orthologous region.

### Defining three-way orthologous regions

First, mouse orthologous regions of rat tsDMRs (MO-tsDMRs) were identified (Mapping orthologous regions). Second, HO-tsDMRs were determined utilizing the same pipeline. Third, human orthologous regions of MO-tsDMRs (HO of MO-tsDMRs) were determined utilizing the same pipeline. Fourth, HO-tsDMRs were compared to the HO of MO-tsDMRs. For a HO-tsDMR and a HO of MO-tsDMR that corresponded to the same rat tsDMR, we required them to have ≥90% overlap. Fifth, the HO-tsDMRs that meet the description in step 4 were obtained and the corresponding MO-tsDMRs and rat tsDMRs are considered as 3-way orthologous regions.

### Defining epigenetic conservation

Epigenetic conservation was defined as the maintenance of a methylation pattern at certain loci within a certain tissue among the species that were examined. For instance, if a 500 bp region in rat was defined as a rat blood tsDMR (i.e., the region is hypomethylated in rat blood and methylated in rat brain and rat sperm) and if the orthologous region of this rat blood tsDMR in mouse and human showed the same tissue-specific methylation pattern (hypomethylated in blood and hypermethylated in brain and sperm), then this rat blood tsDMR was defined as an EC blood tsDMR in rat, mouse, and human.

In mouse and human, the methylation data available for all 6 samples were methylCRF/WGBS, thus single CpG resolution whole-genome DNA methylation was used to identify tsDMRs. For orthologous regions of rat tsDMRs, only the orthologous regions with CpG dinucleotides were retained. For each orthologous region, average methylation was calculated as the sum of methylation level at individual CpGs divided by the number of CpGs in that region. The definition of epigenetic conservation was based on a method described in Sproul et al. 2012 where sites were defined as methylated if their methylation level was greater than 0.7, unmethylated if they had average methylation values less than 0.3, and otherwise intermediately methylated [76]. We assigned a categorical value to each of the three methylation statuses: unmethylated = 0, intermediately methylated = 1, methylated = 2. Based on this categorization, if an orthologous region had a 0 methylation status in the target tissue, and 1 or 2 in the other two tissues, or, if the methylation status was 1 in the target tissue, and 2 in both of the other two tissue types, the region was defined as EC. In all other cases, (for example an orthologous region with a 2 methylation status in the target tissue, and 1 or 2 in the other two tissues) the orthologous region is defined as ENC.

### Calculating distance to the TSS

RefGene annotations were downloaded from the UCSC Genome Browser [77, 78]. The closest TSS to a tsDMR was defined as the TSS with shortest distance to the start of the tsDMR irrespective of strand.

### Genomic features

RepeatMasker annotations, CGIs, and refGene features (including 5’UTRs, exons, and 3’UTRs) were downloaded from the UCSC Genome Browser. Promoters were defined as 2.5 kb around the most 5′ TSS (2 kb upstream and 0.5 kb downstream from TSS) of any refGene record. Intergenic regions were defined as regions between neighboring refGene loci.

### Calculating genetic conservation at tsDMRs

The nine-way phastCons conserved elements file in rn4 format was downloaded from the UCSC Genome Browser. Using bedtools [79], tsDMRs were overlapped with the conserved element track. Genetic conservation was defined when ≥20% of a tsDMR overlapped with elements in the nine-way track; otherwise the tsDMR was defined as genetically not conserved.

### Calculating epigenetic conservation enrichment

Forty six-way human (hg19) vertebrate phyloP scores and 30-way mouse (mm9) vertebrate phyloP scores were downloaded from the UCSC Genome Browser. Since there were no phyloP score files for rat (rn4), we used human vertebrate phyloP scores for the rat-human comparison and mouse vertebrate phyloP scores for the rat-mouse comparison. Each 500 bp window in rat was divided into ten 50 bp windows, and their orthologous regions in mouse and human were determined. For the rat-mouse comparison, a phyloP score was computed for every base from 30 vertebrate genomes, and an average phyloP score was computed for each mouse region orthologous to the rat 50 bp region. The same was done for the rat-human comparison. For each species pair, these genomic segments (orthologous regions of rat tsDMRs) were divided into 100 equal-sized sets with increasing average phyloP scores. The first set with the smallest phyloP scores are defined as the fastest-changing DNA sequences. The last set with the largest phyloP scores are the most conserved which are under purifying selection. In the rat-mouse comparison, a 50 bp rat tsDMR was determined to be EC if the mouse orthologous region was marked by the same DNA methylation pattern across the three tissue types (described in **Defining epigenetic conservation**)**.** For each of the 100 sets, quantitative epigenetic conservation was calculated as the number of rat tsDMRs that were EC between rat and mouse divided by the total number of rat tsDMRs belonging to that set. Rat-human comparisons were done similarly.

### Motif analysis

For each region set of interest, we determined all known motifs that were present within the regions. Motif matching was performed using HOMER [44]. For the rat-mouse comparison, two motif analyses were done: one analysis was of the rat tsDMRs with mouse orthologous regions and the other was of the corresponding mouse orthologous sequences. The top 10 significant motifs (based on *p*-value) within rat tsDMRs were obtained and were used for downstream motif analysis. To determine if these motifs played a role in epigenetic conservation, we further divided the tsDMRs/ orthologous regions into two subsets: the EC tsDMRs and their orthologous regions in mouse/human as well as the ENC tsDMRs and their orthologous regions in mouse/human. We performed HOMER analysis again on the top 10 tissue-specific motifs, and obtained their fold change enrichment over the background among each of the four groups of tsDMRs/ orthologous regions. In order to further determine the contribution of these motifs to the tsDMR conservation across species, we examined the genomic location of each motif occurrence.

## Additional files

Additional file 1: Table S1. Datasets used in this study. **Table S2.** Summary of rat tsDMRs overlapping TEs. **Table S3.** Enrichment of TE subfamilies overlapping rat sperm tsDMRs. **Table S4.** Summary of three-way orthologous rat tsDMRs. **Table S5.** Rat EC and ENC tsDMR distance from the nearest TSS to the start of the tsDMR. **Table S6.** Genomic coordinates and motif sequences of the TF binding sites shown in the five examples. (DOCX 121 kb)

Additional file 2: Figure S1. Genome-wide methylation distribution across the three tissue types in the three species. **Figure S2.** Genomic distribution and locations of rat tsDMRs. **Figure S3.** Views of TE copies in LTR subfamilies that are significantly enriched for rat sperm tsDMRs. **Figure S4.** Percentage of rat regions in different genomic features. **Figure S5.** Genomic distribution of epigenetically conserved and non-conserved tsDMRs in rat and human. **Figure S6.** Genomic distribution of mouse and human orthologous regions of epigenetically conserved and non-conserved tsDMRs. **Figure S7.** Genomic distribution of epigenetically conserved and non-conserved tsDMRs associated with promoters in rat and human. **Figure S8.** Histone modification signatures at human orthologous regions of rat tsDMRs. **Figure S9.** Epigenetically conserved and epigenetically non-conserved rat intergenic tsDMRs show distinct genetic conservation. **Figure S10.** Epigenetic conservation status of tsDMRs shows distinct genomic distributions. **Figure S11.** Histone modification signatures at mouse and human orthologous regions of rat tsDMRs. **Figure S12.** Epigenetic conservation status of tsDMRs shows distinct genetic conservation. **Figure S13.** Epigenetic conservation status of tsDMRs shows distinct transcription factor binding. (DOC 6997 kb)

Additional file 3. Supplementary Text. (DOCX 105 kb)

## Abbreviations

CGIs: CpG islands
DHSs: DNase I hypersensitive sites
DNase I: Deoxyribonuclease I
EC: Epigenetically conserved
ENC: Epigenetically non-conserved
GC: Genetically conserved
GEO: Gene Expression Omnibus
GNC: Genetically non-conserved
HO of MO-tsDMRs: Human orthologous regions of MO-tsDMRs
HO-tsDMRs: Human orthologous regions of rat tsDMRs
IRF: Interferon regulatory TF
MeDIP-seq: Methylated DNA immunoprecipitation followed by sequencing
MO-tsDMRs: Mouse orthologous regions of rat tsDMRs
MRE-seq: Methyl-sensitive restriction enzyme digestion followed by sequencing
RH EC: Epigenetically conserved rat tsDMRs in rat and human, but not mouse
RM EC: Epigenetically conserved rat tsDMRs in rat and mouse, but not human
RMH EC: Epigenetically conserved rat tsDMRs across rat, mouse, and human
RMH ENC: Rat tsDMRs that are epigenetically not conserved in either mouse or human
RPKM: Reads Per Kilobase of transcript per Million mapped reads
TEs: Transposable elements
TFBSs: Transcription factor binding sites
TFs: Transcription factors
Three-way orthologous regions: Regions that were orthologous in rat, mouse, and human
tsDMRs: Tissue-specific differentially methylated regions
TSS: Transcription start site
WGBS: Whole-genome bisulfite sequencing
