# Supplementary material for: Tissue-specific DNA methylation is conserved across human, mouse, and rat, and driven by primary sequence conservation
Source: BMC Genomics. 2017 Sep 12;18:724. doi: 10.1186/s12864-017-4115-6 (PMC5596466; doi:10.1186/s12864-017-4115-6)
Supplement: Supplementary file 1 — Datasets used in this study. Table S2. Summary of rat tsDMRs overlapping TEs. Table S3. Enrichment of TE subfamilies overlapping rat sperm tsDMRs. Table S4. Summary of three-way orthologous rat tsDMRs. Table S5. Rat EC and ENC tsDMR distance from the nearest TSS to the start of the tsDMR. Table S6. Genomic coordinates and motif sequences of the TF binding sites shown in the five examples. (DOCX 121 kb) [file 12864_2017_4115_MOESM1_ESM.docx]

## Supplementary Tables

## Supplementary Table 1: Datasets used in this study. (A) MeDIP-seq datasets. (B) MRE-seq datasets. (C) Whole-genome bisulfite sequencing datasets. (D) Histone modification ChIP-seq datasets.

**A: MeDIP-seq datasets**

| **Species** | **Tissue type** | **Sample description** | **GEO ID (SRA)** | **Data producer** |
| --- | --- | --- | --- | --- |
| Rat | Blood | Whole Blood | GSM1551778 | Wang lab |
|  | Brain | Whole Brain | GSM1551779 | Wang lab |
|  | Sperm | Sperm | GSM1551780 | Wang lab |
| Mouse | Blood | Whole Blood | GSM1551781 | Wang lab |
|  | Brain | Cultured Neuron | GSM1299110 | Wang lab |
| Human | Blood | Blood PBMC, TC003 | GSM543023 | Public |
|  | Brain | Prefrontal cortex grey matter | SRX019264 | Public |

**B: MRE-seq datasets**

| **Species** | **Tissue type** | **Sample description** | **GEO ID (SRA)** | **Data producer** |
| --- | --- | --- | --- | --- |
| Rat | Blood | Whole Blood | GSM1551782 | Wang lab |
|  | Brain | Whole Brain | GSM1551783 | Wang lab |
|  | Sperm | Sperm | GSM1551784 | Wang lab |
| Mouse | Blood | Whole Blood | GSM1551785 | Wang lab |
|  | Brain | Cultured Neuron | GSM1299111 | Wang lab |
| Human | Blood | Blood PBMC, TC003 | GSM543009 | Public |
|  | Brain | Prefrontal cortex grey matter | SRX019323 | Public |

**C: Whole Genome Bisulfite sequencing datasets**

| **Species** | **Tissue type** | **Sample description** | **GEO ID (SRA)** | **Data producer** |
| --- | --- | --- | --- | --- |
| Mouse | Sperm | Mature Sperm | GSE49623 | Public |
| Human | Sperm | Sperm | GSE30340 | Public |

**D: Histone modification ChIP-seq datasets**

| **Species** | **Tissue type** | **Sample description** | **GEO ID (SRA)** | **Data producer** |
| --- | --- | --- | --- | --- |
| Mouse | Blood | Thymus, Histone H3K4me1 | GSM1000102 | Public |
|  |  | Thymus, Histone H3K4me3 | GSM1000101 | Public |
|  |  | Thymus, Histone H3K27ac | GSM1000103 | Public |
|  |  | Thymus, Histone H3K27me3 | GSM1000144 | Public |
|  |  | Thymus, ChIP-Seq Input | GSM1000204 | Public |
|  |  | Thymus, ChIP-Seq Input | GSM918705 | Public |
|  | Brain | Whole Brain, Histone H3K4me1 | GSM1000096 | Public |
|  |  | Whole Brain, Histone H3K4me3 | GSM1000095 | Public |
|  |  | Whole Brain, Histone H3K27ac | GSM1000094 | Public |
|  |  | Whole Brain, Histone H3K27me3 | GSM1000143 | Public |
|  |  | Whole Brain, ChIP-Seq Input | GSM918752 | Public |
|  |  | Whole Brain, ChIP-Seq Input | GSM1000098 | Public |
|  | Sperm | Testis, Histone H3K4me1 | GSM1000078 | Public |
|  |  | Testis, Histone H3K4me3 | GSM1000079 | Public |
|  |  | Testis, Histone H3K27ac | GSM1000081 | Public |
|  |  | Testis, Histone H3K27me3 | GSM1000145 | Public |
|  |  | Testis, ChIP-Seq Input | GSM918751 | Public |
|  |  | Testis, ChIP-Seq Input | GSM1000203 | Public |
| Human | Blood | Blood PBMC, H3K4me1 | GSM1127143 | Public |
|  |  | Blood PBMC, H3K4me3 | GSM1127126 | Public |
|  |  | Blood PBMC, H3K27ac | GSM1127145 | Public |
|  |  | Blood PBMC, H3K27me3 | GSM1127130 | Public |
|  |  | Blood PBMC, ChIP-Seq Input | GSM1127151 | Public |
|  | Brain | Hippocampus middle, H3K4me1 | GSM916039 | Public |
|  |  | Hippocampus middle, H3K4me3 | GSM916040 | Public |
|  |  | Hippocampus middle, H3K27ac | GSM916035 | Public |
|  |  | Hippocampus middle, H3K27me3 | GSM916038 | Public |
|  |  | Hippocampus middle, ChIP-Seq Input | GSM916040 | Public |

**Supplementary Table 2: Summary of rat tsDMRs overlapping TEs.**

| **Rat (rn4)** | **Rat tsDMRs** | **Rat tsDMRs  overlapping TEs** |
| --- | --- | --- |
| Blood | 5506 | 577 (10.48%) |
| Brain | 6861 | 331 (4.82%) |
| Sperm | 40971 | 8018 (19.57%) |
| *Random | 40000 | 13648 (34.12%) |

*Random refers to a set of forty thousand 500bp rat regions randomly selected from the rat genome.

**Supplementary Table 3: Enrichment of TE subfamilies overlapping rat sperm tsDMRs.**

| **TE subfamily**** | **TE family** | **TE class** | **TE tsDMRs in sperm** | **TE tsDMRs in background*** | **Chi-square p-value** | **Fold change over background** |
| --- | --- | --- | --- | --- | --- | --- |
| RMER3D2 | ERVK | LTR | 60 | 1 | 5.93E-19 | 88.616 |
| RMER17B | ERVK | LTR | 71 | 4 | 2.12E-20 | 26.215 |
| RLTR17 | ERVK | LTR | 177 | 10 | 5.20E-50 | 26.142 |
| RMER21A | ERV1 | LTR | 27 | 2 | 7.80E-08 | 19.939 |
| RMER3D3 | ERVK | LTR | 23 | 2 | 1.30E-06 | 16.985 |
| RLTR20B1 | ERVK | LTR | 40 | 4 | 1.08E-10 | 14.769 |
| RMER3D4 | ERVK | LTR | 28 | 3 | 1.42E-07 | 13.785 |
| LTR48 | ERV1 | LTR | 9 | 1 | 0.00651954 | 13.292 |
| RNERVK22 | ERVK | LTR | 17 | 2 | 9.01E-05 | 12.554 |
| MER57D | ERV1 | LTR | 7 | 1 | 0.026758367 | 10.338 |

*Background refers to the forty thousand 500bp regions randomly chosen from the rat genome. **TE subfamilies listed in this table have a Chi-square test p-value < 0.05 and a fold change > 10.

**Supplementary Table 4: Summary of three-way orthologous rat tsDMRs**

| **All tsDMRs** | **Rat tsDMRs** | **Three-way orthologous tsDMRs** |
| --- | --- | --- |
| **Blood** | 5506 | 2859 (52%) |
| ***Blood-matched** |  | 1728 (31%) |
| **Brain** | 6861 | 4746 (69%) |
| ***Brain-matched** |  | 2405 (35%) |
| **Sperm** | 40971 | 16987 (41%) |
| ***Sperm-matched** |  | 12912 (32%) |
| ****Genome random** | 40000 | 10184 (25%) |
|  |  |  |
| **Intergenic tsDMRs** | **Rat tsDMRs** | **Three-way orthologous tsDMRs** |
| **Blood** | 3197 | 1626 (51%) |
| **Blood-matched** |  | 728 (23%) |
| **Brain** | 3056 | 2213 (72%) |
| **Brain-matched** |  | 709 (23%) |
| **Sperm** | 30676 | 11653 (38%) |
| **Sperm-matched** |  | 6873 (22%) |

* Blood-matched regions are the control set that matches the genomic distribution of rat blood tsDMRs. The same is true for Brain-matched and Sperm-matched regions. **Genome random is a set of forty thousand 500bp rat regions that are randomly chosen from the rat genome.

**Supplementary Table 5: Rat EC and ENC tsDMR distance from the nearest TSS to the start of the tsDMR.**

| **Species Comparison** | **Tissue** | **EC Distance* to the TSS (Mean)** | **ENC Distance* to the TSS (Mean)** | **Wilcox p.value**  **(EC < ENC) one-sided** |
| --- | --- | --- | --- | --- |
| **Rat-Mouse** | Blood | 57,641.02 | 73,551.66 | 6.10E-17 |
|  | Brain | 96,375.93 | 78,770.65 | 1 |
|  | Sperm | 125,715.44 | 144,645.01 | 1.85E-77 |
| **Rat-Human** | Blood | 36,322.83 | 80,543.11 | 2.57E-41 |
|  | Brain | 44,221.69 | 99,469.63 | 1.55E-46 |
|  | Sperm | 129,020.98 | 146,713.12 | 4.29E-49 |

* Distances are given as base pair units and are averaged across the full group of EC or ENC tsDMRs.

**Supplementary Table 6: Genomic coordinates and motif sequences of the TF binding sites shown in the five examples.**

GC = Genetically conserved

GNC = Genetically non-conserved

RMH EC = Epigenetically conserved across rat, mouse, and human

RM EC = Epigenetically conserved in rat and mouse, but not human

RH EC = Epigenetically conserved in rat and human, but not mouse

| **Category** | **Motif** | **Gene** | **Species** | **tsDMR/** **orthologous region coordinates** | **Motif sequence** |
| --- | --- | --- | --- | --- | --- |
| GC  RMH EC | Lhx3 | Sez6 | Rat | chr10: 64007000-64007500 | CCCTAATTAT, CTAATTATAG, CTGTAATTAG, GTAATTAGCC |
|  |  |  | Mouse | chr11: 77779006-77779523 | CCCTAATTAT, CTAATTATAG, CTGTAATTAG, GTAATTAGCC |
|  |  |  | Human | chr17: 27294813-27295316 | CCCTAATTAT, CTAATTATAG, CTGTAATTAG, GTAATTAGCC |
|  |  |  |  |  |  |
| GNC  RMH EC | Erg | Erg | Rat | chr11:35756000-35756500 | GCAGGAAGCA  [84bp]  ***−−CTGAGCAC*** |
|  |  |  | Mouse | chr16:95676600-95677127 | GCAGGAAGCA  [84bp] ***−−CTGAGCAC*** |
|  |  |  | Human | chr21:39865918-39866537 | CCAGG***G***AGCA  [84bp]  CAGGAAGGAA |
|  |  |  |  |  |  |
| GC  RM EC | Fli1 | Skap1 | Rat | chr10:85389000-85389500 | GAAGGAAGTG |
|  |  |  | Mouse | chr11:96503962-96504467 | GAAGGAAGTG |
|  |  |  | Human | chr17:46321342-46321848 | GAAGGAA***A***TG |
|  |  |  | Dog | chr9:27844513-27845025 | GAAGGAA***A***TG |
|  |  |  |  |  |  |
| GC  RH EC | Fli | Cd6 | Rat | chr1:213289000-213289500 | TCAGGAAGCC  [ 357bp]  AACTTCCT***CT*** |
|  |  |  | Mouse | chr19:10862127-10862638 | TCAGGA***G***GCC  [357bp ]  CAC***CTCT***GC***A*** |
|  |  |  | Human | chr11:60793251-60793801 | TC**^ACATGGAAAGG^**AGGA***G***GGC  [357bp]  CACTTCCGCC |
|  |  |  |  |  |  |
| Between tsDMRs  TF turnover | Fli1 | Irf2 | Rat | chr16:48707500-48708000 | CACTTCCTGC  [6kb]  CCTTT***G***CTAA, ***TATCTCGCCC*** |
|  |  |  | Mouse | chr8:47896625-47897124 | CACTTCCTGG  [6kb]  CCT***CGG***CTCC,  ***TGTCCTGCCT*** |
|  |  |  | Human | chr4:185336158-185336700 | CACTT **^ACTTC^** CCTGG  [6kb]  CCTTTCCGGA,  CGCTTCCTCT |

Sequences highlighted in red are the DNA sequences that are not TF binding motifs. Nucleotides highlighted in red are the mutations/InDels that disrupted the TF binding motifs.
