# Supplementary material for: Tissue-specific DNA methylation is conserved across human, mouse, and rat, and driven by primary sequence conservation
Source: BMC Genomics. 2017 Sep 12;18:724. doi: 10.1186/s12864-017-4115-6 (PMC5596466; doi:10.1186/s12864-017-4115-6)
Supplement: Supplementary file 2 — Genome-wide methylation distribution across the three tissue types in the three species. Figure S2. Genomic distribution and locations of rat tsDMRs. Figure S3. Views of TE copies in LTR subfamilies that are significantly enriched for rat sperm tsDMRs. Figure S4. Percentage of rat regions in different genomic features. Figure S5. Genomic distribution of epigenetically conserved and non-conserved tsDMRs in rat and human. Figure S6. Genomic distribution of mouse and human orthologous regions of epigenetically conserved and non-conserved tsDMRs. Figure S7. Genomic distribution of epigenetically conserved and non-conserved tsDMRs associated with promoters in rat and human. Figure S8. Histone modification signatures at human orthologous regions of rat tsDMRs. Figure S9. Epigenetically conserved and epigenetically non-conserved rat intergenic tsDMRs show distinct genetic conservation. Figure S10. Epigenetic conservation status of tsDMRs shows distinct genomic distributions. Figure S11. Histone modification signatures at mouse and human orthologous regions of rat tsDMRs. Figure S12. Epigenetic conservation status of tsDMRs shows distinct genetic conservation. Figure S13. Epigenetic conservation status of tsDMRs shows distinct transcription factor binding. (DOC 6997 kb) [file 12864_2017_4115_MOESM2_ESM.doc]

**Supplementary Figures**

**
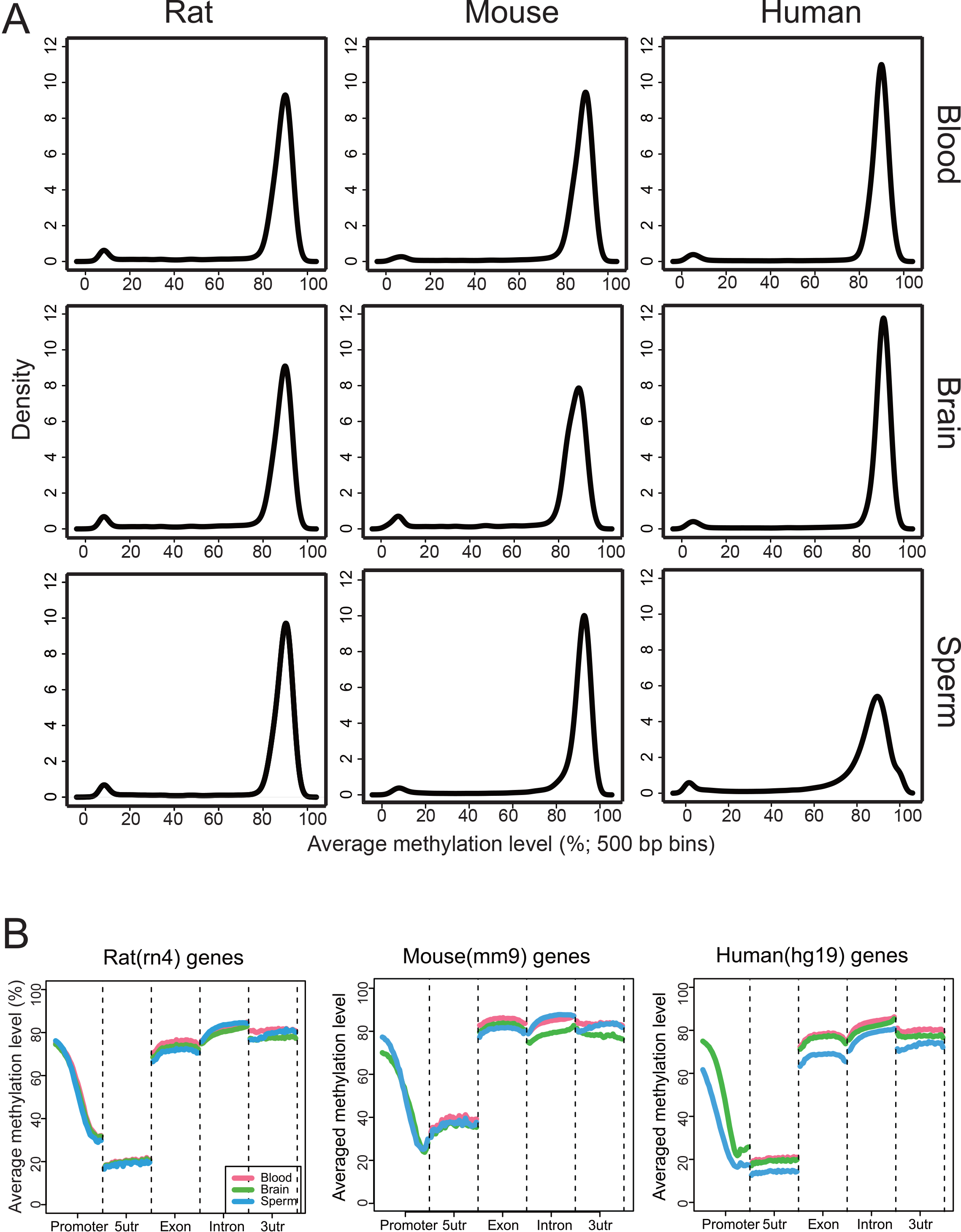
**

**Supplementary Figure 1. Genome-wide methylation distribution across the three tissue types in the three species**. (**A**) Distribution of the average methylation level in 500bp non-overlapping windows in the rat genome and their orthologous regions in the mouse and human genome for each tissue type. Each panel indicates the methylation distribution of a specific tissue type (indicated to the right of the panels) in a specific species (indicated above the panels). The x-axis represents the methylation level (%) from 0 to 100, with 0 meaning unmethylated and 100 meaning methylated. For mouse and human sperm samples, WGBS data was used to calculate the average methylation at each mouse or human orthologous regions of the rat 500bp regions since MeDIP-seq and MRE-seq data was not available. For the other seven samples, methylCRF was used to estimate the methylation level at single CpG resolution. (**B**) Average methylation level across different gene features. Gene features were annotated using the UCSC gene annotations. For every gene, each gene feature, e.g., promoters, was divided into 30 sub-regions of equal size and the average methylation score (WGBS for mouse and human sperm and methylCRF for the other seven samples) was calculated for each sub-region. The methylation scores were averaged across all genes and plotted for each sub-region for each gene feature.


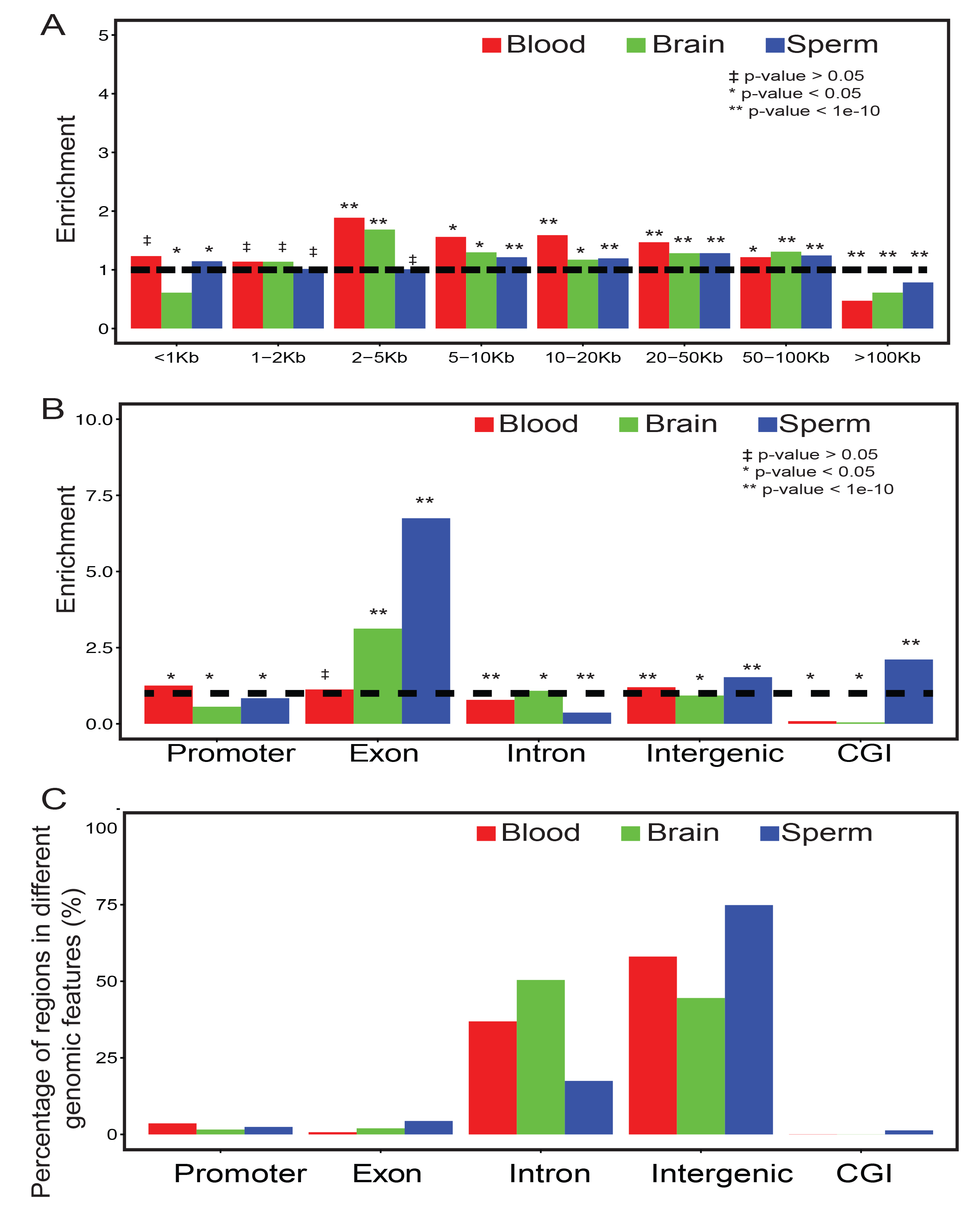


**Supplementary Figure 2. Genomic distribution and locations of rat tsDMRs**. (**A**) Distribution of the distance between rat tsDMRs and the nearest TSS. An annotation-matched control set was selected for each tissue based on the genomic distribution of the tsDMRs in each tissue type. The horizontal dashed black line denotes no enrichment over the background. The y-axis represents the enrichment of rat tsDMRs located within different distance range groups relative to the background distribution. A Chi-square test was performed to obtain p-values. P-values were corrected for multiple testing using the Benjamini–Hochberg FDR method. (**B**) Genomic distribution of rat tsDMRs. The y-axis represents the enrichment of rat tsDMRs associated with different genomic features relative to the background genomic distribution. A Chi-square test was performed to obtain p-values. P-values were corrected for multiple testing using the Benjamini–Hochberg FDR method. (**C**) Percentage of rat tsDMRs located in different genomic features.

**
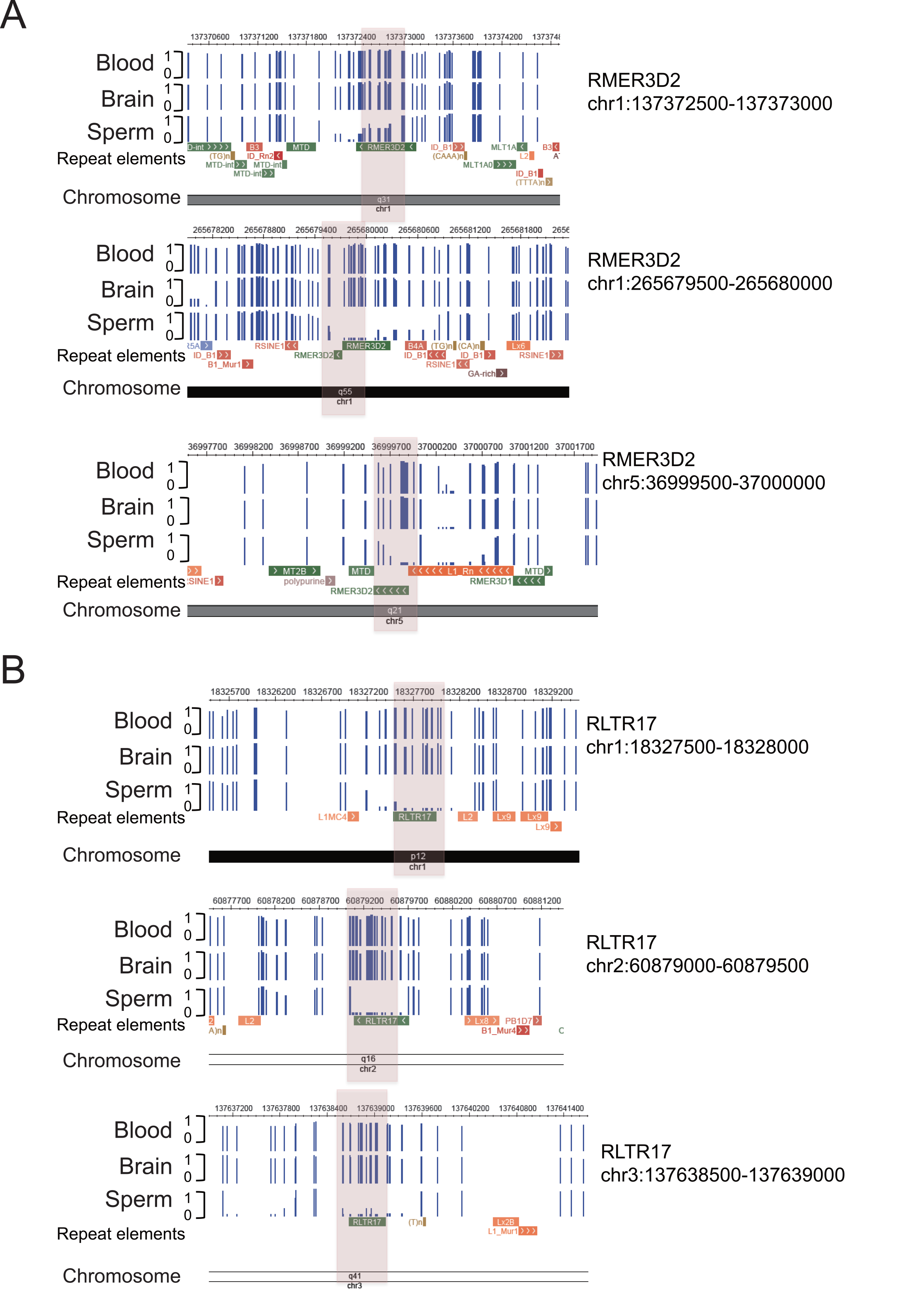
**

**Supplementary Figure 3. Views of TE copies in LTR subfamilies that are significantly enriched for rat sperm tsDMRs.** Displayed tracks include: methyl tracks for DNA methylation for rat blood, brain, and sperm, the repeatMasker track, and the chromosome track. The rat sperm tsDMRs are highlighted in pink rectangles. The height of the bars in the methyl tracks indicates the methylation level. (**A**) Genome browser views of three RMER3D2 subfamily copies that show sperm specific hypomethylation. (**B**) Genome browser views of three RLTR17 subfamily copies that show sperm specific hypomethylation.

**
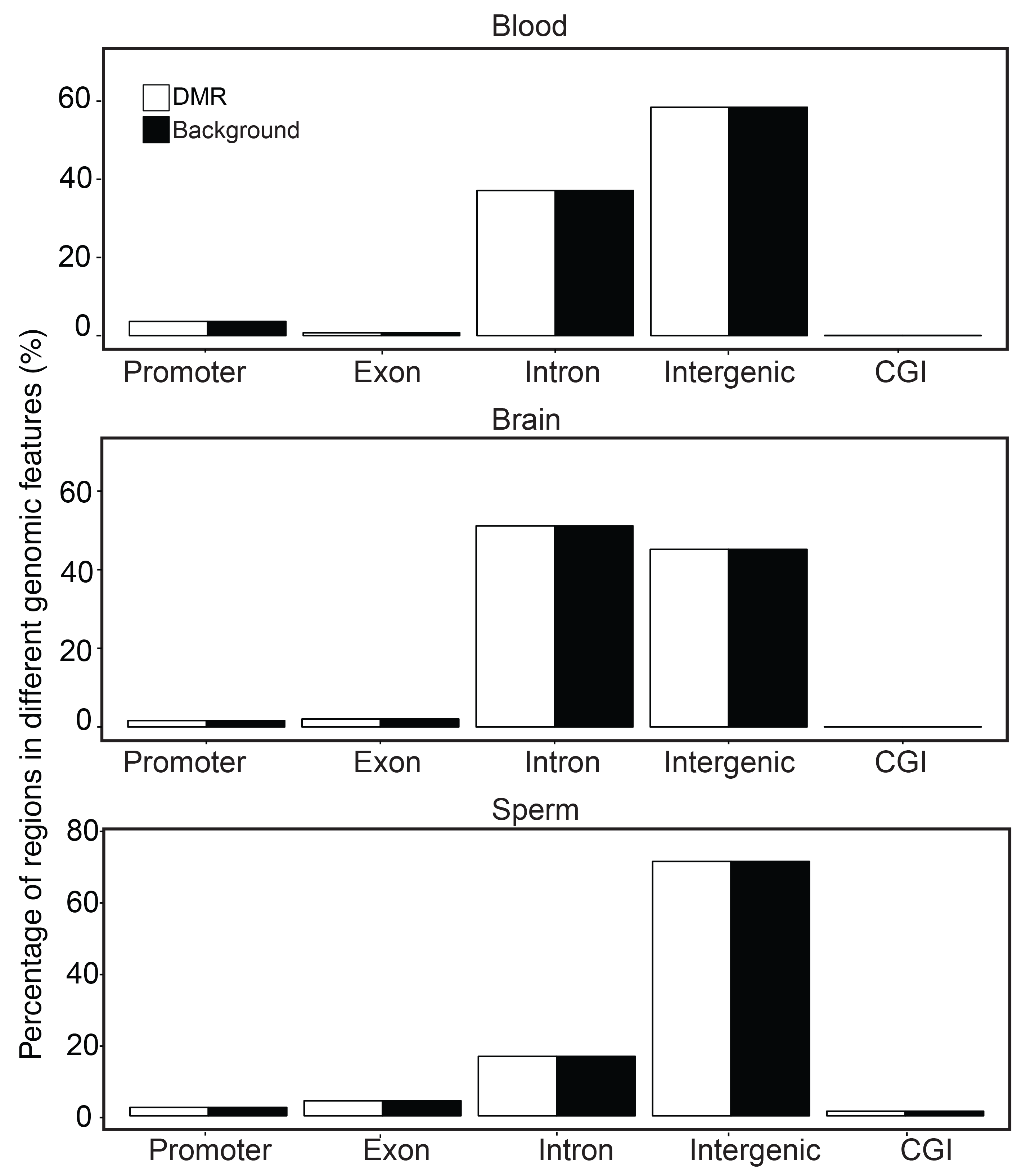
**

**Supplementary Figure. 4. Percentage of rat regions in different genomic features.** Percentage of rat tsDMRs (in white) vs. genomic distribution-matched control set (in black) in different genomic features in blood (*top panel*), brain (*middle panel*), and sperm (*bottom panel*).

**
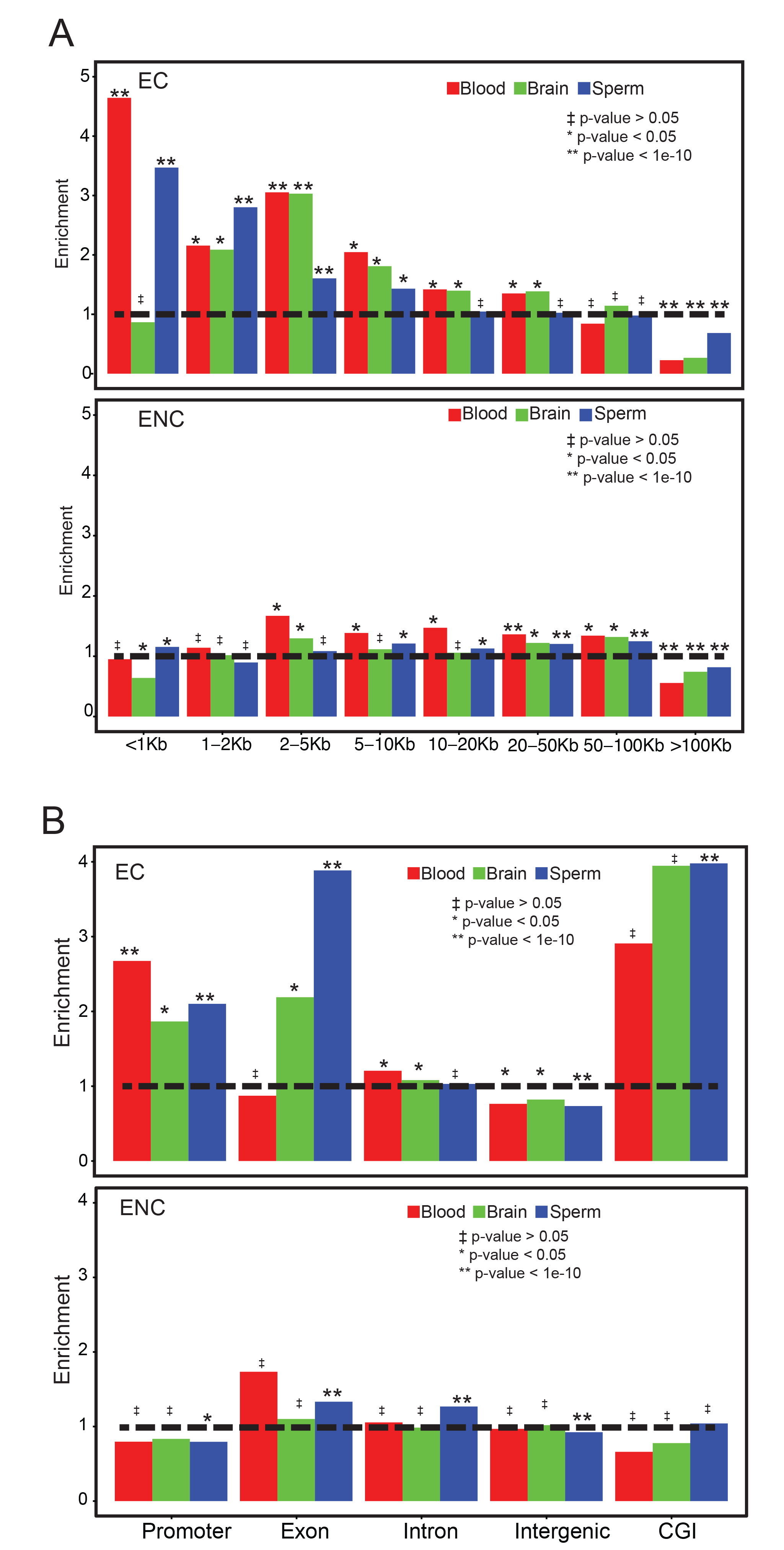
**

**Supplementary Figure 5. Genomic distribution of epigenetically conserved and non-conserved tsDMRs in rat and human.** (**A**) Distribution of the distance between rat tsDMRs that are epigenetically conserved in human (*top* row) and epigenetically non-conserved in human (*bottom* row) to the nearest TSS. An annotation-matched control set was selected for each tissue based on the genomic distribution of the tsDMRs in each tissue type. The y-axis represents the fold enrichment of rat tsDMRs over the background. The horizontal dashed black line denotes no enrichment over the background. (**B**) Genomic distribution of rat tsDMRs that are epigenetically conserved in human (*top* row) and epigenetically non-conserved in human (*bottom* row). The background regions were chosen the same way as described in (**A**). A Chi-square test was performed to obtain p-values. P-values were corrected for multiple testing using the Benjamini–Hochberg FDR method.

**
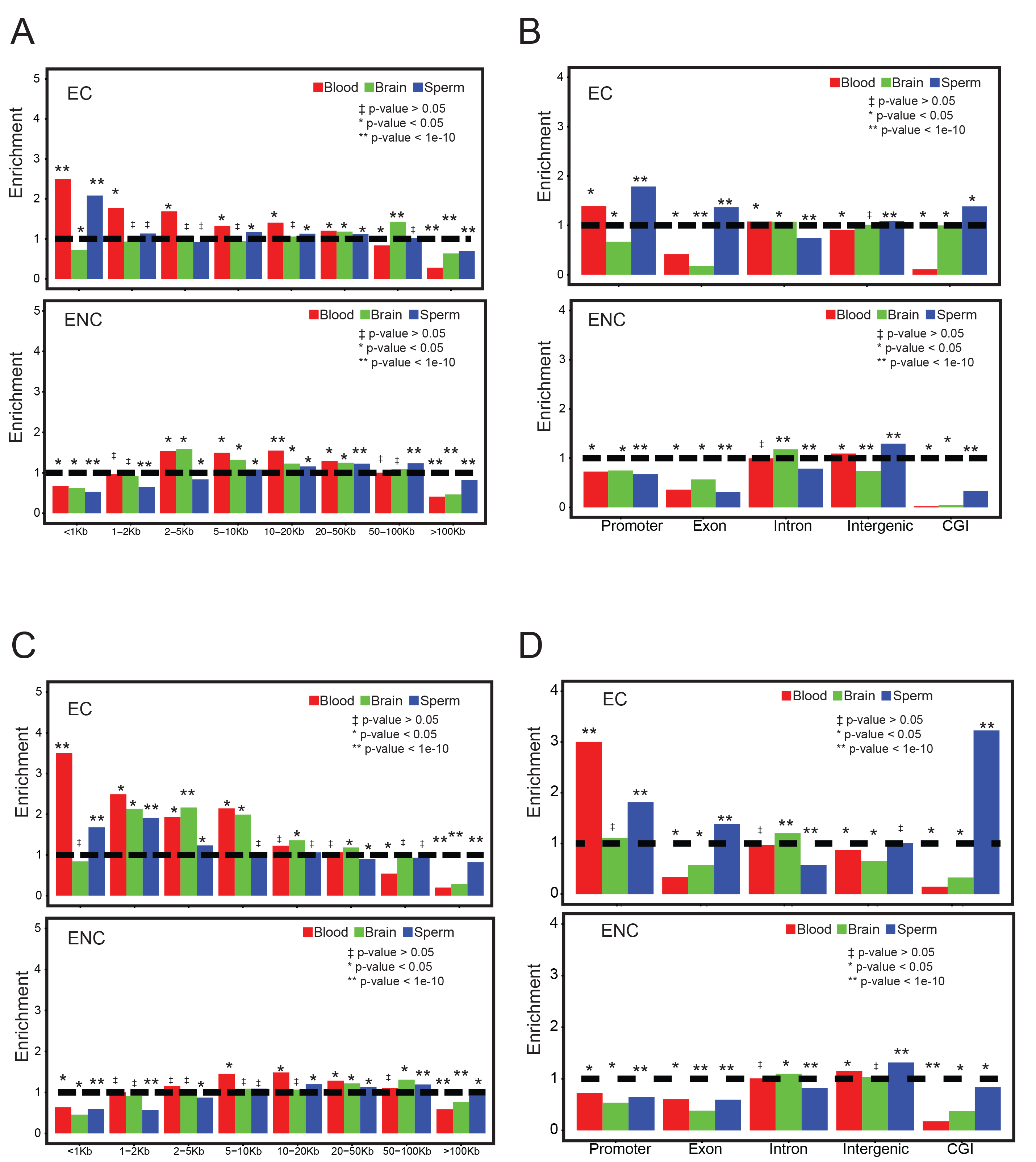
**

**Supplementary Figure 6. Genomic distribution of mouse and human orthologous regions of epigenetically conserved and non-conserved tsDMRs.** (**A**) Distribution of the distance between mouse orthologous regions of rat tsDMRs that are epigenetically conserved in mouse (*top* row) and epigenetically non-conserved in mouse (*bottom* row) to the nearest TSS. The y-axis represents the fold enrichment of mouse orthologous regions of rat tsDMRs over the background. The horizontal dashed black line denotes no enrichment over the background. (**B**) Genomic distribution of mouse orthologous regions of rat tsDMRs that are epigenetically conserved in mouse (*top* row) and epigenetically non-conserved in mouse *(bottom* row). In panels (**C**) and (**D**), human genomic regions that were orthologous to the rat genome were used instead of mouse genomic regions. A Chi-square test was performed to obtain p-values. P-values were corrected for multiple testing using the Benjamini–Hochberg FDR method.

**
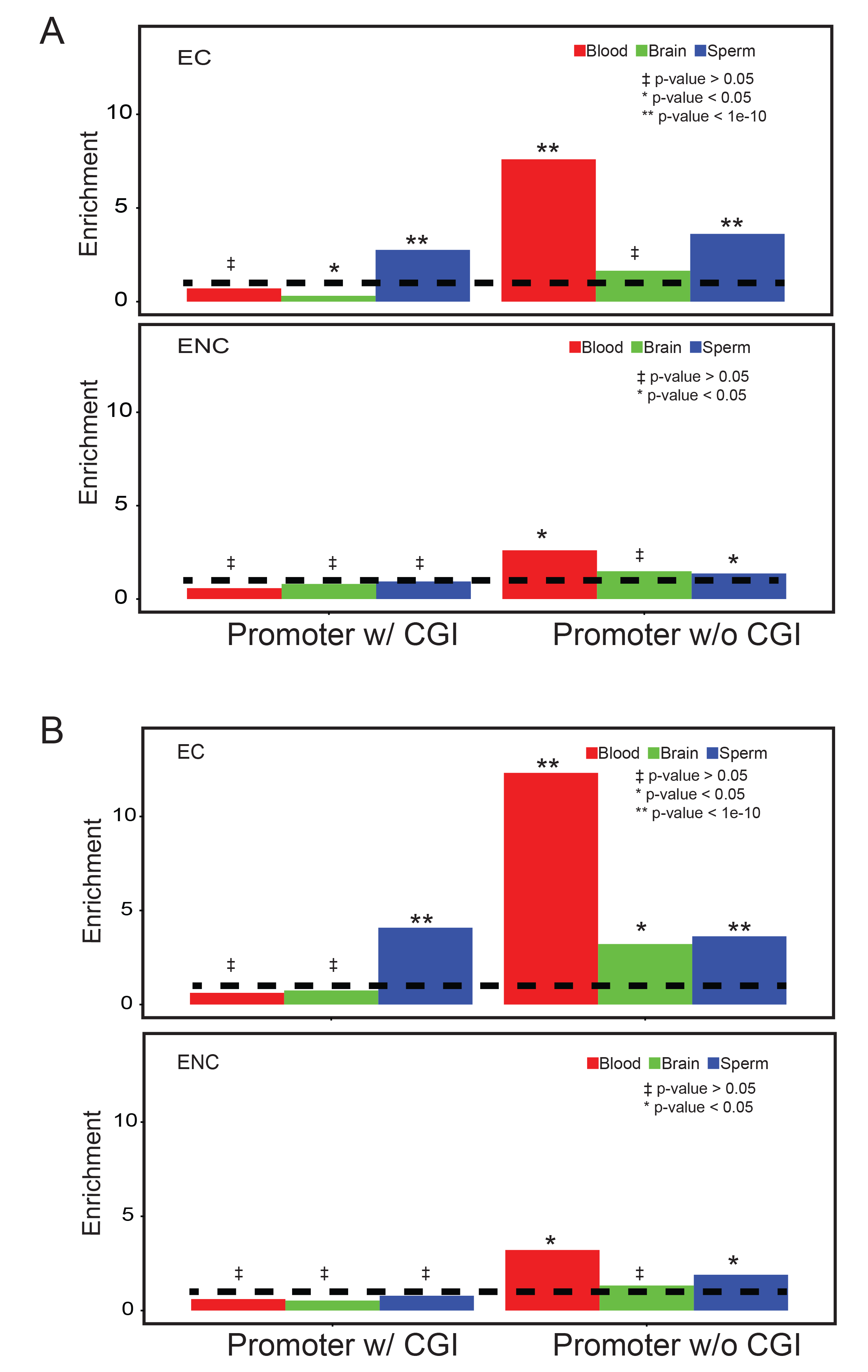
**

**Supplementary Figure 7. Genomic distribution of epigenetically conserved and non-conserved tsDMRs associated with promoters in rat and human.** (**A**) Genomic distribution of rat promoter tsDMRs (CpG-promoters vs. non-CpG promoters) that are epigenetically conserved in mouse (*top* row) and epigenetically non-conserved in mouse (*bottom* row). (**B**) Genomic distribution of rat promoter tsDMRs (CpG-promoters vs. non-CpG promoters) that are epigenetically conserved in human (*top* row) and epigenetically non-conserved in human (*bottom* row). A Chi-square test was performed to obtain p-values. P-values were corrected for multiple testing using the Benjamini–Hochberg FDR method.

**
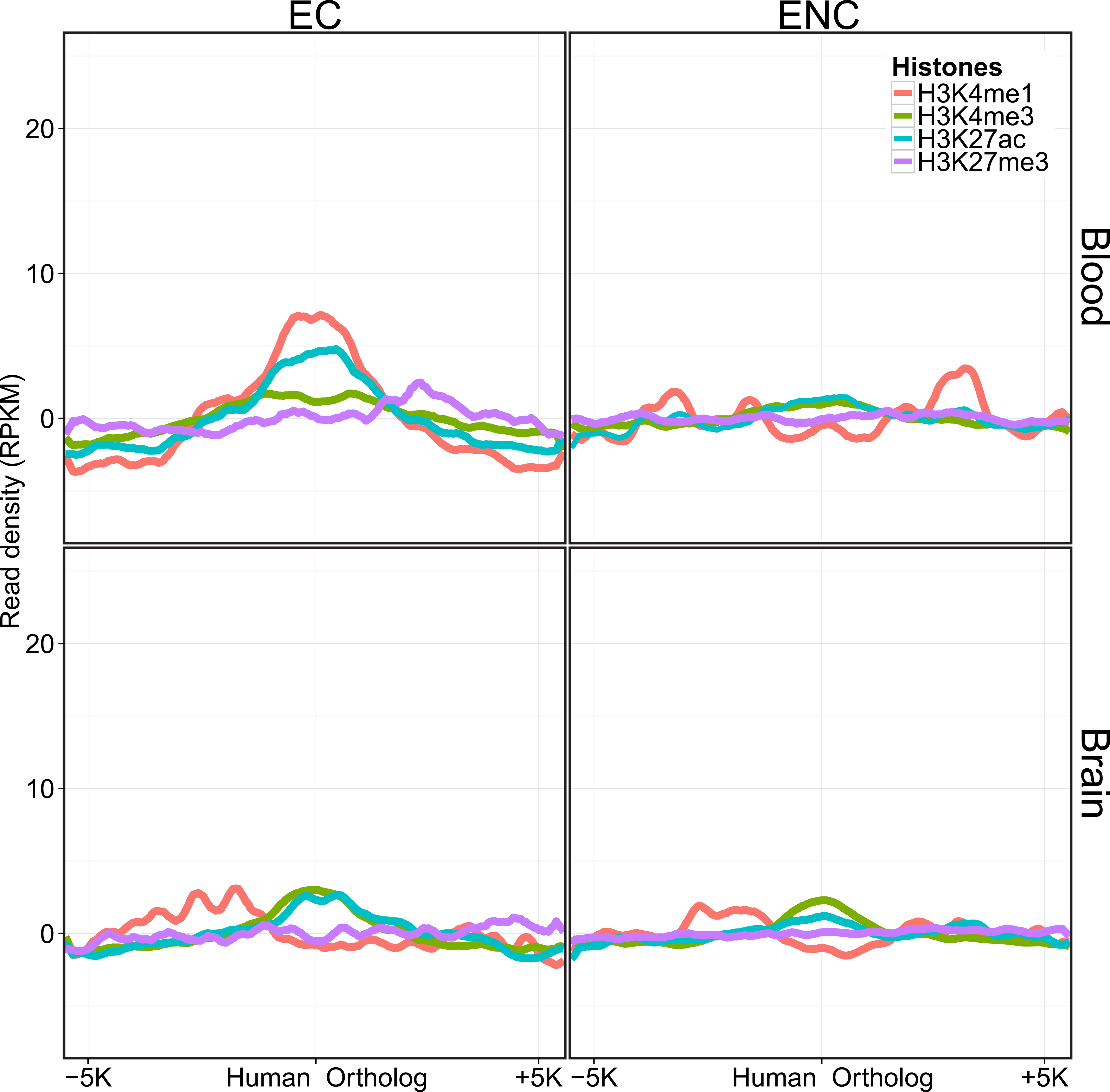
**

**Supplementary Figure 8. Histone modification signatures at human orthologous regions of rat tsDMRs.** Average histone modification signal density at 50-bp resolution over a 10-kb window centered on human orthologous regions of rat tsDMRs that are epigenetically conserved (*left* column) and epigenetically non-conserved (*right* column) in human blood (*top* row), and human brain (*bottom* row).





**Supplementary Figure 9. Epigenetically conserved and epigenetically non-conserved rat intergenic tsDMRs show distinct genetic conservation.** (**A**) Percentage of rat intergenic tsDMRs that are genetically conserved. The number of genetically conserved intergenic tsDMRs is indicated above the bars for each tissue type. For each tissue type, a genomic annotation matched random control set was chosen. A Chi-square test was performed to obtain p-values. P-values were corrected for multiple testing using the Benjamini–Hochberg FDR method. (**B**) Percentage of EC intergenic rat tsDMRs in mouse that overlap with genetically conserved rat elements (in red) and the percentage of ENC rat intergenic tsDMRs in mouse that overlap with genetically conserved rat elements (in green). A Chi-square test was performed to obtain the p-values. P-values were corrected for multiple testing using the Benjamini–Hochberg FDR method. (**C**) Comparison of phastCons score distributions of EC rat intergenic tsDMRs and ENC rat intergenic tsDMRs in mouse. A Wilcoxon test was performed to obtain p-values. P-values were corrected for multiple testing using the Benjamini–Hochberg FDR method.

**
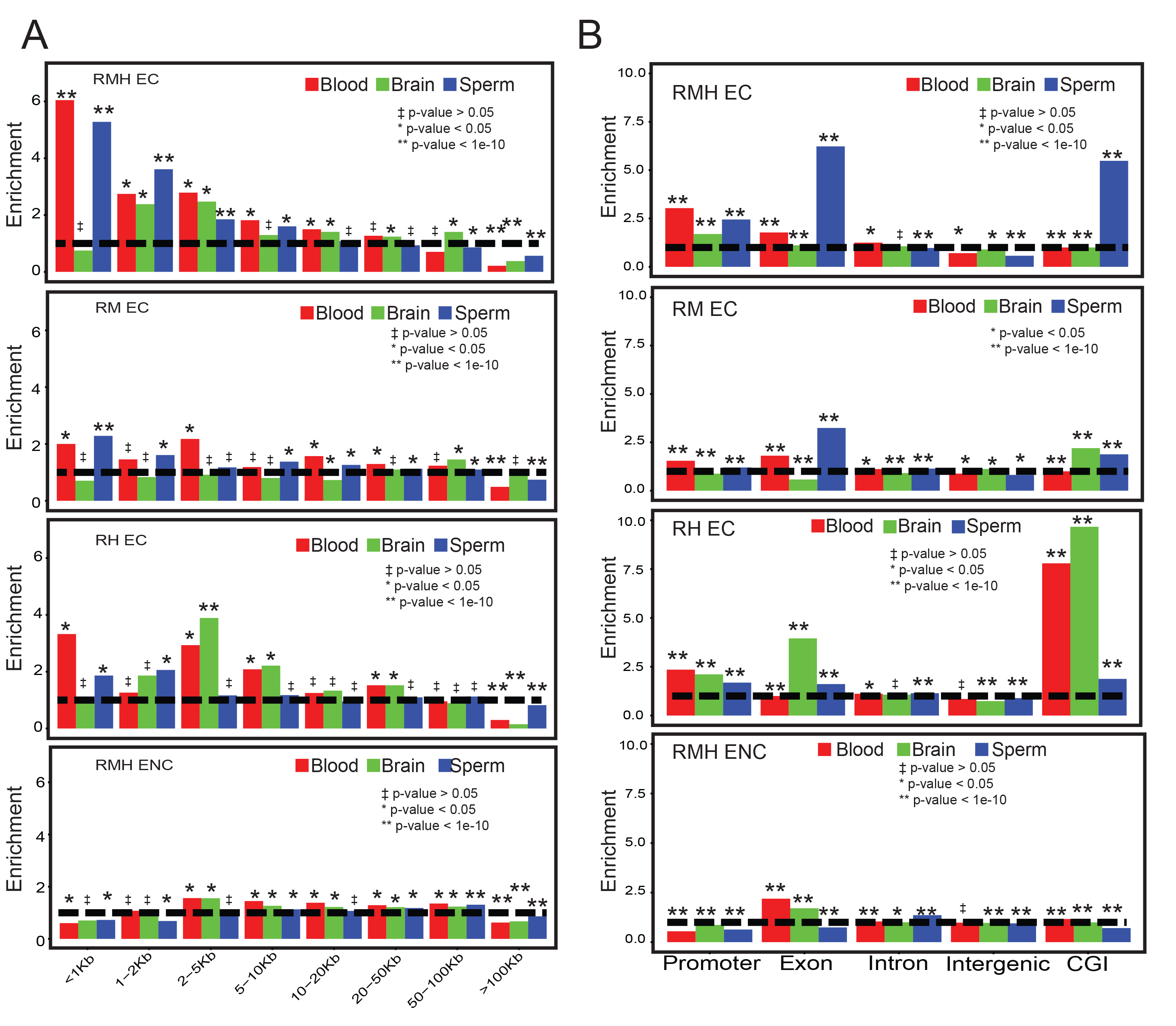
**

**Supplementary Figure 10. Epigenetic conservation status of tsDMRs shows distinct genomic distributions.** The four epigenetic conservation statuses are: Group 1: rat tsDMRs that are EC across rat, mouse, and human (RMH EC); Group 2: rat tsDMRs that are EC in rat and mouse, but not in human (RM EC); Group 3: rat tsDMRs that are EC in rat and human, but not in mouse (RH EC); Group 4: rat tsDMRs that are ENC in either mouse or human (RMH ENC). (**A**) Distribution of the distance between rat tsDMRs in each of the four epigenetic conservation categories and the nearest TSS. The four panels from *top* to *bottom* represent the distribution of distance in Group 1, Group2, Group 3, and Group 4, respectively. The horizontal dashed black line denotes no enrichment over the background. An annotation-matched control set was selected for each tissue based on the genomic distribution of the tsDMRs in each tissue type. (**B**) Genomic distribution of rat tsDMRs in each of the four epigenetic conservation categories. The order of the panels is the same as (A). The background regions were chosen the same way as described in (**A**). A Chi-square test was performed to obtain p-values. P-values were corrected for multiple testing using the Benjamini–Hochberg FDR method.


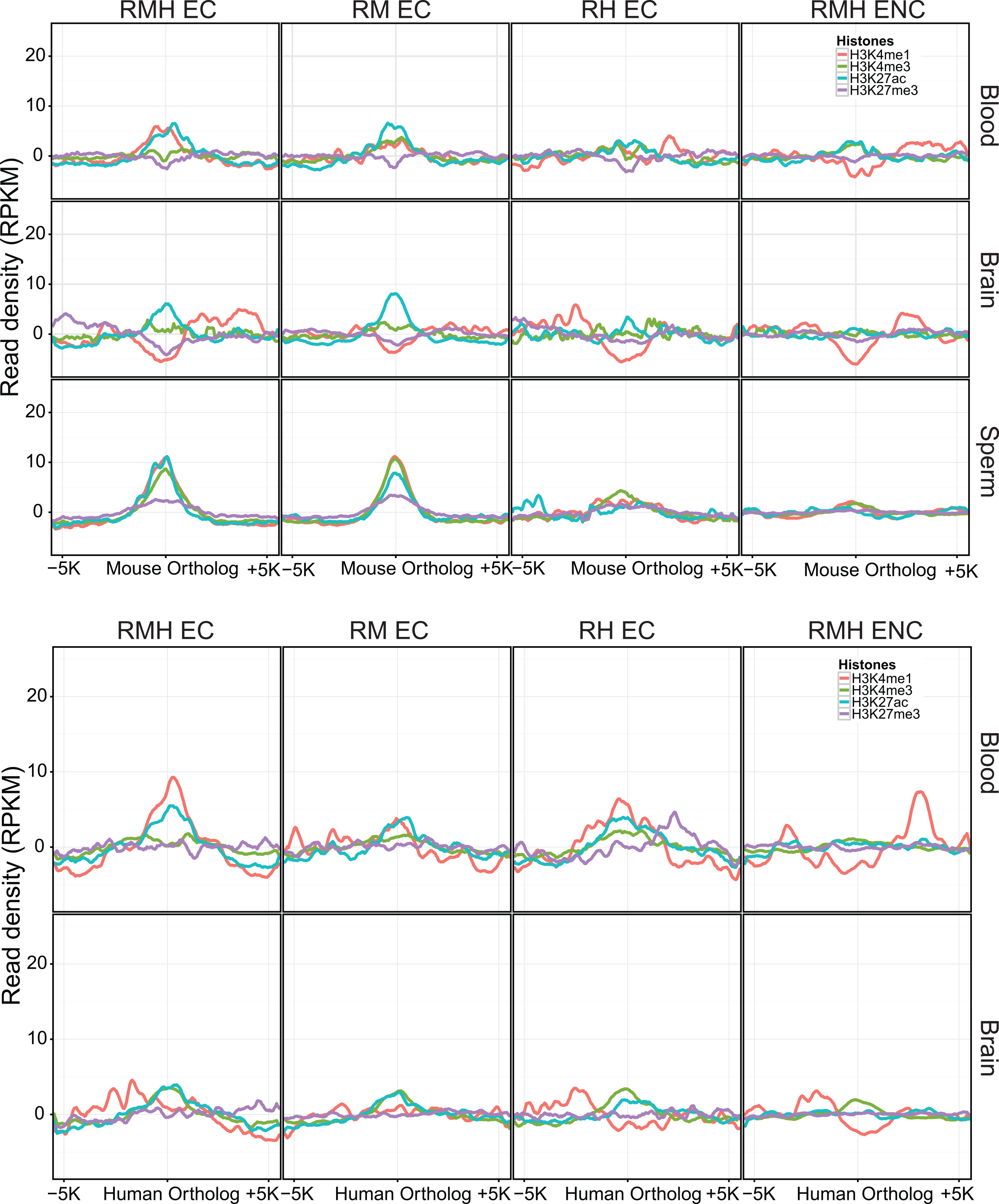


**Supplementary Figure 11.** **Histone modification signatures at mouse and human orthologous regions of rat tsDMRs**. The four epigenetic conservation groups were defined in **Supplementary Figures, Figure 10**. Upper panels: average histone modification signal density at 50-bp resolution over a 10-kb window centered on mouse orthologous regions of rat tsDMRs in each of the four epigenetic conservation categories (RMH EC, column 1; RM EC, column 2; RH EC, column 3; and RMH ENC, column 4), in mouse blood (*first* row), mouse brain (*second* row) and mouse sperm (*third* row). Lower panels: average histone modification signal density at 50-bp resolution over a 10-kb window centered on human orthologous regions of rat tsDMRs in each of the four epigenetic conservation categories (RMH EC, column 1; RM EC, column 2; RH EC, column 3; and RMH ENC, column 4), in human blood (*fourth* row) and human brain (*fifth* row).


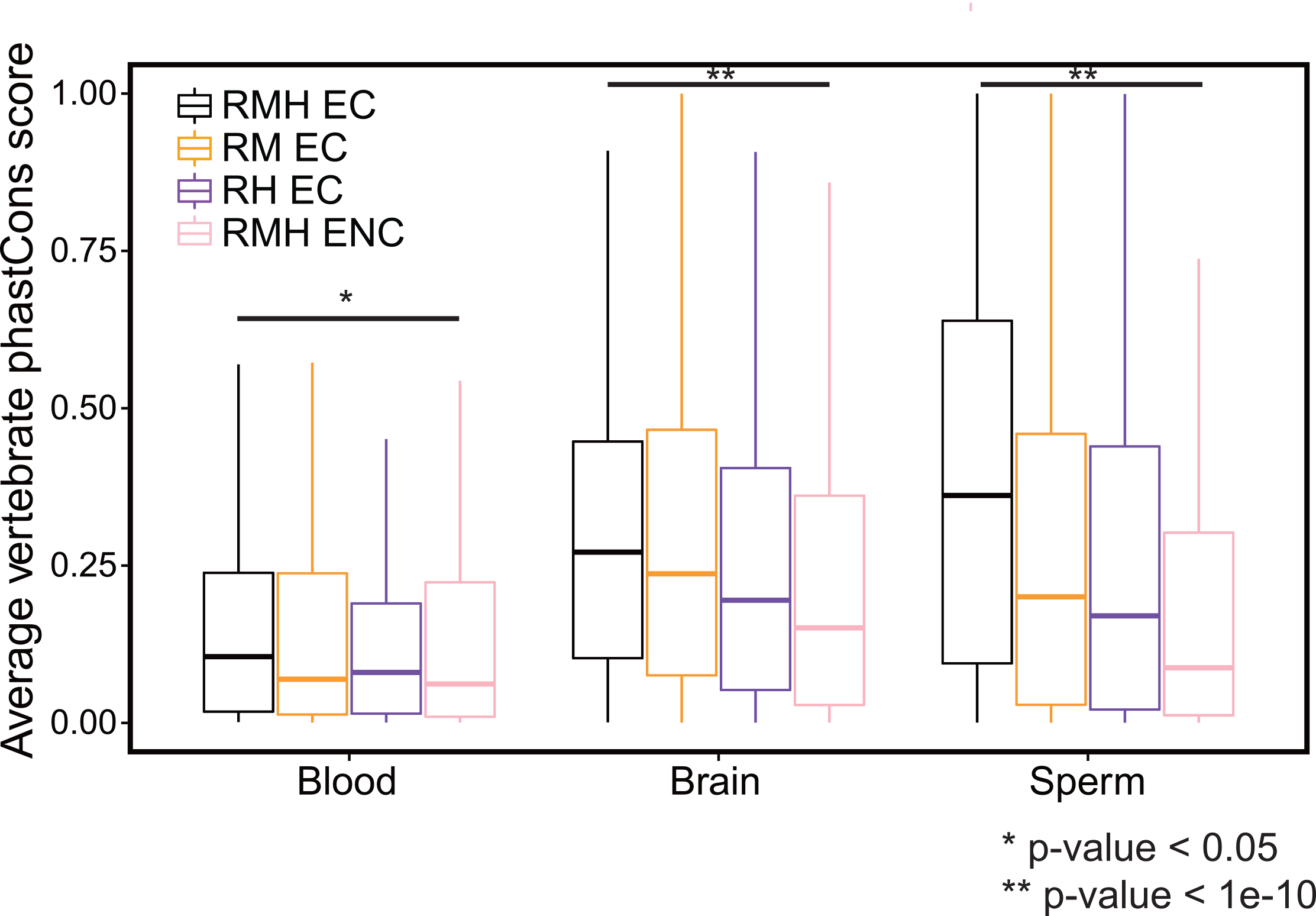


**Supplementary Figure 12.** **Epigenetic conservation status of tsDMRs shows distinct genetic conservation.** The four epigenetic conservation groups were defined as described in **Supplementary Figures, Figure 10**. PhastCons score distributions of rat tsDMRs in each of the four epigenetic conservation categories. A Wilcoxon-test was performed to obtain p-values for the RMH-EC vs. RMH-ENC comparison. P-values were corrected for multiple testing using the Benjamini–Hochberg FDR method.


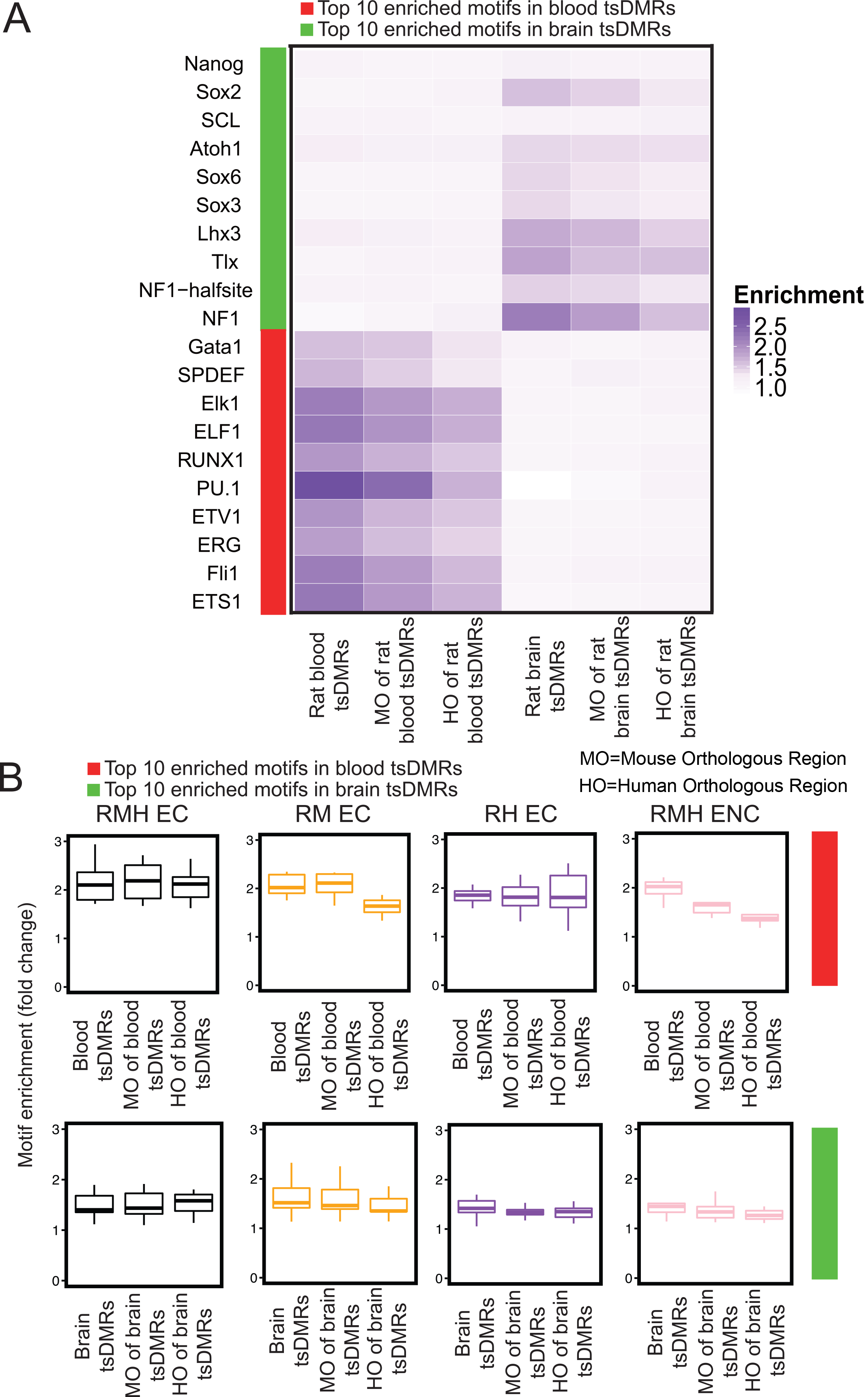


**Supplementary Figure 13.** **Epigenetic conservation status of tsDMRs shows distinct transcription factor binding.** The four groups were defined in **Supplementary Figures, Figure 10.** (**A**) Heatmap representing the enrichment of transcription factor binding motifs for the tsDMRs identified in each tissue and for the mouse and human orthologous regions of these tsDMRs. Each row represents a motif, and the corresponding transcription factor for selected motifs are labeled on the left. (**B**) Motif enrichment (fold change) of the top 10 TF motifs in rat blood tsDMRs and the corresponding mouse and human orthologous regions (*top* row) and rat brain tsDMRs and the corresponding mouse and human orthologous regions (*bottom* row), for each epigenetic conservation category (RMH EC, *first* column; RM EC, *second* column; RH EC, *third* column; and RMH EC, *fourth* column).
