# Supplementary material for: Tissue-specific DNA methylation is conserved across human, mouse, and rat, and driven by primary sequence conservation
Source: BMC Genomics. 2017 Sep 12;18:724. doi: 10.1186/s12864-017-4115-6 (PMC5596466; doi:10.1186/s12864-017-4115-6)
Supplement: Supplementary file 3 — Supplementary Text. (DOCX 105 kb) [file 12864_2017_4115_MOESM3_ESM.docx]

**Supplementary Text**

**Supplementary Methods**

**TE analysis on rat sperm tsDMRs**

RepeatMasker annotations were downloaded from the UCSC Genome Browser [1,2]. The selection of TE-derived tsDMRs was based on the following criteria: 1) ≥ 50% overlap of the tsDMR with a repeat element and 2) the repeat elements belonged to one of the following four classes: SINE, LINE, LTR, or DNA. Forty thousand 500bp regions were randomly chosen from the rat genome and the overlap between the random set and the repeat elements was defined using the same criteria as described above. The enrichment score for each subfamily with respect to tsDMRs was calculated as:

$${ES}_{subfamily\_i}=\frac{ns\_dmr/nt\_dmr}{ns\_random/nt\_random}$$

Where *ns_dmr* is the number of tsDMRs that overlapped subfamily *i*; *nt_dmr* is the number of tsDMRs that overlapped any TE; *ns_random* is the number of random regions that overlapped subfamily *i*; *nt_random* is the number of random regions that overlapped any TE. The significant TE subfamilies hypomethylated in rat sperm were defined as the ones with a Chi-square test p-value < 0.05, and an enrichment score > 10.

**Example M&M pairwise comparison commands**

**# Count MeDIP-seq and MRE-seq in 500bp bins for sample1**

countMeDIPbin(file.Medipsite=<MeDIP-seq1 bed file>, file.blacklist=<black_list>, file.bin=<CpG count for the 500bp bins>, file.CNV=NULL, writefile=<MeDIP count output file1>, reportfile=<MeDIP count output report file1>, binlength=500)

countMREbin(file.Medipsite=<MRE-seq1 bed file>, file.blacklist=<black_list>, file.bin=<CpG count for the 500bp bins>, file.CNV=NULL, cutoff=0.05, writefile=<MRE count output file1>, reportfile=<MRE count output report file1>, binlength=500)

**# Count MeDIP-seq and MRE-seq in 500bp bins for sample2**

countMeDIPbin(file.Medipsite=<MeDIP-seq2 bed file>, file.blacklist=<black_list>, file.bin=<CpG count for the 500bp bins>, file.CNV=NULL, writefile=<MeDIP count output file2>,reportfile=<MeDIP count output report file2>, binlength=500)

countMREbin(file.Medipsite=<MRE-seq2 bed file>, file.blacklist=<black_list>, file.bin=<CpG count for the 500bp bins>, file.CNV=NULL, cutoff=0.05, writefile=<MRE count output file2>,reportfile=<MRE count output report file2>, binlength=500)

**# Calculate methylMnM (M&M) p-value**

d_f = c(<MeDIP count output file1>, < MeDIP count output file2>, < MRE count output file1>, < MRE count output file2>)

MnM.test(file.dataset=d_f,chrstring=NULL,file.cpgbin=<CpG count for the 500bp bins>,file.mrecpgbin=<MRE CpG count for the 500bp bins, 4 enzymes>, writefile=<M&M p-value output>, reportfile=<M&M p-value report>, mreratio=3/7,method='XXYY', psd=2,mkadded=1,a=1e-16,cut=100,top=500)

**# Calculate q-value**

MnM.qvalue(<M&M p-value output>, <M&M q-value output>)

**# Select DMRs**

q = read.table(<M&M q-value output>,header=T)

dmr = MnM.selectDMR(q,up=1.45,down=1/1.45,q.value=1e-5,cutoff='q-value', quant=0.9)

write.table(dmr,file=<M&M q-value output selected>, sep='\t', quote=F, row.names=F)

**Supplementary Results**

**Sperm tsDMRs were enriched for the LTR class**

There were considerably more tsDMRs in sperm than the other tissue types examined. A close examination of sperm tsDMRs revealed that 8,018 (20%) overlapped TEs (**Supplementary Tables, Table 2**), which is significantly higher than the overlap between TEs and the tsDMRs identified in the other two tissue types. Several TE subfamilies were significantly enriched for hypomethylated sperm tsDMRs (**Supplementary Tables, Table 3**). The most enriched subfamily was RMER3D2, which belongs to the ERVK family of the LTR class. This subfamily had 60 individual copies located in sperm tsDMRs, corresponding to an 89-fold enrichment compared to the background. Three individual copies located at chr1: 137372500-137373000, chr1:265679500-265680000, and chr5:36999500-37000000, respectively are shown in **Supplementary Figures, Fig. 3A**. Another enriched subfamily was RLTR17, which was discovered 177 times in the sperm tsDMRs, corresponding to a 26-fold enrichment compared to background. Three individual copies belonging to the RLTR7 subfamily, located at chr1:18327500-18328000, chr2:60879000-60879500 and chr3:137638500-137639000, respectively, are shown in **Supplementary Figures, Fig. 3B**.

**Supplementary References**

1. Kent WJ, Sugnet CW, Furey TS, Roskin KM, Pringle TH, Zahler AM, et al. The Human Genome Browser at UCSC. Genome Res. 2002;12:996–1006.

2. Meyer LR, Zweig AS, Hinrichs AS, Karolchik D, Kuhn RM, Wong M, et al. The UCSC Genome Browser database: extensions and updates 2013. Nucleic Acids Res. 2013;41:D64–9.
